# (Arg)_9_-SH2 superbinder: a novel promising anticancer therapy to melanoma by blocking phosphotyrosine signaling

**DOI:** 10.1186/s13046-018-0812-5

**Published:** 2018-07-05

**Authors:** An-dong Liu, Hui Xu, Ya-nan Gao, Dan-ni Luo, Zhao-feng Li, Courtney Voss, Shawn S. C. Li, Xuan Cao

**Affiliations:** 1 Department of Medical Genetics, School of Basic Medicine, Tongji Medical College, Huazhong University of Science and Technology, Wuhan, 430030 China; 2 Ultrastructural Pathology Laboratory, Department of Pathology, School of Basic Medicine, Tongji Medical College, Huazhong University of Science and Technology, Wuhan, 430030 China; 3 Department of Biochemistry, Schulich School of Medicine and Dentistry, Western University, London, ON N6A 5C1 Canada; 4 Institute for Brain Research, Huazhong University of Science and Technology, Wuhan, 430030 China; 5 Key Laboratory of Neurological Disease of National Education Ministry, Tongji Medical College, Huazhong University of Science and Technology, Wuhan, 430030 China; 6 Key Laboratory for Drug Target Researches and Pharmacodynamic Evaluation of Hubei Province, Wuhan, 430030 China

**Keywords:** Melanoma, Phosphorylated tyrosine proteins, SH2 superbinder, Cell penetrating peptides, Drug resistance

## Abstract

**Background:**

Melanoma is a malignant tumor with high misdiagnosis rate and poor prognosis. The bio-targeted therapy is a prevailing method in the treatment of melanoma; however, the accompanying drug resistance is inevitable. SH2 superbinder, a triple-mutant of the Src Homology 2 (SH2) domain, shows potent antitumor ability by replacing natural SH2-containing proteins and blocking multiple pY-based signaling pathways. Polyarginine (Arg)_9_, a powerful vector for intracellular delivery of large molecules, could transport therapeutic agents across cell membrane. The purpose of this study is to construct (Arg)_9_-SH2 superbinder and investigate its effects on melanoma cells, expecting to provide potential new approaches for anti-cancer therapy and overcoming the unavoidable drug resistance of single-targeted antitumor agents.

**Methods:**

(Arg)_9_ and SH2 superbinder were fused to form (Arg)_9_-SH2 superbinder via genetic engineering. Pull down assay was performed to identify that (Arg)_9_-SH2 superbinder could capture a wide variety of pY proteins. Immunofluorescence was used to detect the efficiency of (Arg)_9_-SH2 superbinder entering cells. The proliferation ability was assessed by MTT and colony formation assay. In addition, wound healing and transwell assay were performed to evaluate migration of B16F10, A375 and A375/DDP cells. Moreover, apoptosis caused by (Arg)_9_-SH2 superbinder was analyzed by flow cytometry-based Annexin V/PI. Furthermore, western blot revealed that (Arg)_9_-SH2 superbinder influenced some pY-related signaling pathways. Finally, B16F10 xenograft model was established to confirm whether (Arg)_9_-SH2 superbinder could restrain the growth of tumor.

**Results:**

Our data showed that (Arg)_9_-SH2 superbinder had the ability to enter melanoma cells effectively and displayed strong affinities for various pY proteins. Furthermore, (Arg)_9_-SH2 superbinder could repress proliferation, migration and induce apoptosis of melanoma cells by regulating PI3K/AKT, MAPK/ERK and JAK/STAT signaling pathways. Importantly, (Arg)_9_-SH2 superbinder could significantly inhibit the growth of tumor in mice.

**Conclusions:**

(Arg)_9_-SH2 superbinder exhibited high affinities for pY proteins, which showed effective anticancer ability by replacing SH2-containing proteins and blocking diverse pY-based pathways. The remarkable ability of (Arg)_9_-SH2 superbinder to inhibit cancer cell proliferation and tumor growth might open the door to explore the SH2 superbinder as a therapeutic agent for cancer treatment.

**Electronic supplementary material:**

The online version of this article (10.1186/s13046-018-0812-5) contains supplementary material, which is available to authorized users.

## Background

Melanoma, which can be cured by surgery in the early stage but the prognosis is poor, accounts for about 80% of skin cancer deaths. This is largely because metastatic malignancy is refractory to conventional therapies [1–4]. Gene mutations, mostly BRAF mutations, are present in over half of all human melanomas. Although patients with BRAF-mutant are highly sensitive to small molecule inhibitors, like Vemurafenib and Dabrafenib, most patients display partial responses to clinical treatment, which can eventually worsen conditions or cause death due to drug resistance within 6 to 12 months [5–7]. Moreover, patients that lack gene mutations have few effective therapeutic methods. These patients are associated with unique risk factors, pathophysiologic, clinical, and prognostic features that differ from those related to BRAF tumors [8]. Thus, it is necessary and urgent to address the problems of drug resistance to inhibitors with BRAF-mutant and lacking appropriate therapies for patients without specific mutations.

Src Homology 2 (SH2) domains specifically recognize phosphorylated tyrosine and mediate cell signal transduction [9, 10]. The “superbinder” SH2 domain, with triple-point mutants Thr8Val/Cys10Ala/Lys15Leu, abbreviated SH2 superbinder, which could bind pY-containing peptides with much stronger affinity than natural SH2 domains or traditional anti-pY antibody (4G10) [11, 12]. Conventional drugs are generally directed against a single gene or protein and most of melanoma patients develop drug resistance eventually [13]. SH2 superbinder, strongly binding with diverse pY sites to block related signal transduction pathways, can recognize other sites instead, even if mutation alters the pY site [14, 15]. Therefore, SH2 superbinder can achieve the purpose of killing cancer cells [16]. These advantages might resolve problems of drug resistance due to gene mutation and lack of suitable targeting drugs described above. However, it is urgent to settle the problem of transporting SH2 superbinder across cell membrane barriers.

Cell penetrating peptides (CPPs), having ability to translocate across the plasma membranes, are confined to short sequences of less than 20 amino acids [17]. CPPs have several advantages over conventional techniques on cellular delivery because they are efficient for a variety of cells, and have a potential therapeutic application [18]. Nona-arginine (Arg)_9_, as one kind of CPPs, has been applied efficaciously to translocate across the cell membrane and deliver large cargo molecules such as peptides, proteins, and oligonucleotides into cells with no severe side effects, which has a prospect of wide application and a development potential in drug delivery [19, 20].

(Arg)_9_-SH2 superbinder (referred as (Arg)_9_-GST SH2 TrM) was conducted by genetic engineering approach. The results validated that (Arg)_9_-SH2 superbinder had strong ability to translocate into cells and played a role in melanoma cells by binding with various pY proteins. Furthermore, (Arg)_9_-SH2 superbinder could suppress proliferation and migration of melanoma cells. Meanwhile, apoptosis caused by (Arg)_9_-SH2 superbinder was observed. Additionally, PI3K/AKT, MAPK/ERK and JAK/STAT pathways were also affected by (Arg)_9_-SH2 superbinder. Moreover, (Arg)_9_-SH2 superbinder could inhibit the growth of tumor in vivo. Above all, (Arg)_9_-SH2 superbinder, which could translocate into cells efficaciously and provide a novel anticancer method for melanoma via capturing multiple pY-containing proteins, might be a potent candidate in the targeted therapy of melanoma.

## Methods

### Construction, expression and purification of GST fusion proteins

Genes encoding the human wild type and triple-mutant Src SH2 domains, from the pEGFP-C3-Src SH2 Wt/TrM plasmids, were subcloned into the pGEX-4 T3 vector for expressing GST-Src SH2 Wt/TrM proteins. Based on pGEX-4 T3-alone or pGEX-4 T3- Src-SH2 Wt/TrM, recombinant plasmids were constructed for expressing GST-(Arg)_9_, GST-Src-Wt/TrM-(Arg)_9_. Wt represented Wild type. Amino acid sequences and primers of all used constructs were listed in Additional file 1: Table S1 and Additional file 2: Table S2 of supplementary materials. As shown in Additional file 1: Table S1, although nona-arginines were put at the end of C-terminus of GST or GST-Src-SH2 Wt/TrM, we termed them as (Arg)_9_-GST or (Arg)_9_-GST-Src-SH2 Wt/TrM.

All amplified products were gel purified using the Gel Band Purification Kit (Beijing ComWin Biotech). The backbone was digested with BamHI (cat. # R3136V) and NotI (cat. # R3189V) in 37 °C for 2 h and the purified PCR products were reacted with the linearized pGEX-4 T3 vector at 37 °C for 30 min. The experiment is using the One Step Cloning Kit (Vazyme, Nanjing), according to the principle of homologous recombination. E*.coli* BL21 containing the expression plasmid was grown in LB broth with 100 μg/ml ampicillin at 37 °C. The expression of GST fusion protein was induced by the addition of isopropyl β-D-thiogalactoside (0.5 mM final concentration), and then incubated at 20 °C for 18 h. The lysis buffer of protein contains 20 mM Tris-HCl (pH 7.0), 50 mM NaCl, 0.5 mM EDTA, 1 mM dithiothreitol (DTT), 1 mM cocktail, and 1 mM PMSF. GST fusion proteins were purified from bacterial cell lysates by glutathione-agarose beads. After sonication, cell lysates were cleared by centrifugation at 9500 rpm for 30 min, prior to mixing with glutathione-agarose beads. After rotating at 4 °C for 3 h, proteins could be eluted and collected. The protein concentration in the cell homogenates was quantified with BCA Protein Assay Kit. Immediately prior to their use in biological assays, protein purity was verified by SDS-PAGE using Coomassie brilliant blue staining intensity.

### Cell lines and cell culture

B16F10 melanoma cells (no metastasis variant mouse melanoma), A375 (BRAF mutation) were purchased from the American Type Culture Collection (ATCC, Manassas, VA). The cisplatin (DDP)-resistant subline A375/DDP was established with continuous exposure of the parental A375 cells to increasing concentrations of cisplatin, ranging from 2 nM to 4 μM for about 6 months. The drug-resistant cells were maintained in DMEM containing 4 μM cisplatin. All cells were cultured in DMEM medium supplemented with 10% FBS and 100 U/mL penicillin- streptomycin and were maintained in a humid atmosphere with 5% CO2 at 37 °C.

### Glutathione s-transferase pull down assay and western blot

For GST pull down assay, GST fusion proteins were expressed in *E. coli* BL21 (DE3). Cells were treated with phosphatase inhibitor sodium pervanadate (0.5 mM) for 10 min at 37 °C before harvesting. Then, cells were lysed in ice-cold lysis buffer (0.5% NP-40, 50 mM Hepes (pH 7.4), 1 mM magnesium chloride, 150 mM KCl, and the complete protease inhibitor cocktail). For immunoprecipitation and western blot (immunoblot), cells were lysed on ice in lysis buffer (1% NP-40, 50 Mm Tris-HCl (pH 7.4), 150 mM NaCl, 2 mM EDTA, 50 mM NaF, 10% glycerol, and the complete protease inhibitor cocktail). The supernatant was gathered after centrifugation at 12,000 g for 15 min. Protein A/G agarose (Thermo Fisher) and Glutathione Sepharose beads (GE Healthcare) were used for the immunoprecipitation and GST pull down assays, respectively. Protein concentrations were quantified by BCA method. The proteins were separated by a 10% SDS-polyacrylamide gel and eleco-transferred onto PVDF membranes (Millipore), which were incubated in 5% skim milk for 1 h at room temperature. Primary antibodies against EGFR(CST#4267), Grb2(CST#3972), pERK1/2(CST#4370), pSTAT3(CST#4113), pAKT(CST#4060), AKT(CST#9272), ERK1/2(CST#4695), STAT3(CST#4904), pY antibody (Abcam EPR16871), GAPDH(CST#5174), Bax(ABclonal#A12009) and Bcl2(ABclonal#A11025) were diluted at 1:1000 and then incubated with the membranes overnight at 4 °C. Membranes were washed three times for 10 min and incubated with a 1:5000 dilution of HRP-conjugated anti-mouse or anti-rabbit antibodies. Blots were washed with TBST three times and developed with the ECL system; the membranes were exposed to ChemiDoc MP Imager (BIO-RAD). The band densities were normalized relative to the relevant GAPDH with Image J software.

### Immunofluorescence

1 × 10^4^ cells were seeded in a 12-well plate and cultured for 24 h. Cells were incubated with proteins with different time and concentrations. After washing with cold washing buffer, cells were then fixed in 4% formaldehyde at room temperature for 1 h, and then were permeabilized with 0.5% Triton X-100 for 20 min. After rinsing in PBS, cells were treated with rhodamine phalloidin for 30 min and then incubated with DAPI for 5 min at RT. Samples were imaged by a fluorescence microscope (Olympus, Japan). The images were analyzed by Image J software.

### MTT assay

Cells collected in the logarithmic phase were plated into 96-well plates (3–5 × 10^3^ cells/well). On the following day, add GST fusion proteins into the cell culture medium. After incubating for different time, 10 μL of 5 g/L MTT solutions (Sigma) were added into each well and incubated for 4 h, and then incubated with 100 μL DMSO for another 15 min. The optical absorbance was measured at the wavelength of 570 nm. Different treatment time and concentrations have been shown in the figure legends.

### CCK-8 assay

Cells collected in the logarithmic phase were plated into 96-well plates (5 × 10^3^ cells/well). On the following day, add GST fusion proteins into cell culture medium with different concentrations. After incubating for various time, 10 μl CCK-8 solution (Dojindo) was added into each well and incubated for 1-4 h. The optical absorbance was measured at the wavelength of 450 nm.

### Colony formation assay

Cells were trypsinized and plated in 6-well plates (100 cells/well), incubated with GST fusion proteins and counted 14 days after seeding. The colonies were subsequently fixed with 4% formaldehyde and stained with 0.01% crystal violet for 10 min.

### Wound healing assay

Cells were seeded into six-well plates (5 × 10^5^ cells/well). Wounds were then created using the 200 μL pipette tips. The scratched cells were removed by PBS for three times. Cells were then cultured for 16 h incubated in the presence or absence of (Arg)_9_-GST SH2 TrM. Microscopic images were taken with a digital camera at different time points.

### Transwell assay

2 × 10^4^ cells were plated in 200 μL DMEM containing 2% FBS in the upper chamber. The lower chamber was filled with 500 μL completed medium containing 10%FBS. (Arg)_9_-GST SH2 TrM or (Arg)_9_-GST were added to the upper chamber and cells were allowed to migrate for 16 h at 37 °C with 5% CO2. Cells were fixed with 4% formaldehyde for 15 min at room temperature. Then, cells on the upper chamber were moved with a cotton swab. After washing the chambers with ddH_2_O, cells remained on the bottom of the lower chamber were stained with 0.1% crystal violet. The migrated clones were photographed under a microscope. The cell numbers were counted at three different areas.

### Apoptosis analysis

Cells were sedimented by centrifugation, resuspended and fixed in 100 μl binding buffer. Cell density in the cell suspension was adjusted to 2 × 10^3^cells/μl. Subsequently, 5 μl Annexin V-FITC was added to the cell suspension followed by gently vortexing and incubation for 10 min at room temperature in the dark. Thereafter, the cell suspension was incubated with 5 μl Propidiumiodide. All details need to refer to the instructions of kit (Tianjin Sungene Biotech). Cells were analyzed immediately using a FACS flow cytometer (FACS BD Biosciences, Germany) for Annexin V-FITC and Propidiumiodide binding. Dot plots and histograms were analyzed by Flowjo software.

TdT-UTP nick end labeling (TUNEL) assays were performed with one-step TUNEL apoptosis assay kit produced by Beyotime Institute of Biotechnology according to the manufacturer’s instructions. The FITC-labeled TUNEL-positive cells were imaged under a fluorescent microscope (Olympus, Japan). The cells with green fluorescence were defined as apoptotic cells. And images were analyzed by Image J software.

### Animal studies

Xenograft model was established on C57Bl/6 mice (Animal Center of Tongji Medical College, Wuhan), 4–6 weeks old, weighing approximately 18-22 g. All studies involving animals were performed following the National Guides for the Care and Use of Laboratory Animals and approved by the Institutional Animal Care and Use Committee of Tongji Medical College, Huazhong University of Science and Technology.

A suspension of 1 × 10^6^ B16F10 cells (in 100 μl PBS) was subcutaneously injected into the right flank each mouse. After the development of an easily palpable tumor (7–10 days, approximately 5 mm in diameter). Tumor size was measured by using a caliper every 3 days and was calculated by using the following formula: tumor volume = 1/2(width)^2^ × length. Mice were divided into control group (B16F10 cells and PBS injected) and the experimental group (B16F10 cells and (Arg)_9_-GST SH2 TrM treated), *n* = 3 per group. Animals were euthanized when tumor size reached the ethical end point.

### Preparation of PBMCs

Peripheral blood obtained from healthy C57Bl/6 mice was anticoagulated with heparin. PBMC were isolated by Ficoll (Germany) density gradient centrifugation of peripheral venous blood. Cells were washed three times and resuspended in DMEM medium supplemented with 1 mM sodium pyruvate, 1% nonessential amino acids and vitamins, 2 mM L-glutamine, 100 U/ml penicillin-streptomycin and 10% FBS.

### Statistical analysis

The Student’s test was used to test for statistical significance of the differences between the different group parameters. *P* values of less than 0.05 were considered statistically significant.

## Results

### Construction, expression and purification of GST fusion proteins

(Arg)_9_, (Arg)_9_-SH2 Wt/TrM, and SH2 Wt/TrM-alone (without nona-Arg) were subcloned into the pGEX-4 T3 backbone, respectively. The PCR product of SH2 Wt/TrM was 327 bp and the pGEX-4 T3-(Arg)_9_-SH2 Wt/TrM was 5295 bp. DNA gel electrophoresis and sequencing were utilized to confirm the successful construction of pGEX-4 T3-(Arg)_9_-SH2 Wt/TrM (Additional file 3: Figure S1a). Coomassie staining image showed that (Arg)_9_-GST SH2 Wt/TrM and (Arg)_9_-GST were expressed and glutathione-agarose beads were used to purify the expressed proteins (Additional file 3: Figure S1b). The molecular weight of (Arg)_9_-GST, (Arg)_9_-GST SH2 Wt/TrM, and GST SH2 Wt/TrM are about 26 kDa, 36 kDa, and 35 kDa, respectively.

### (Arg)_9_-GST SH2 TrM could effectively bind with pY proteins

**Fig. 1:**
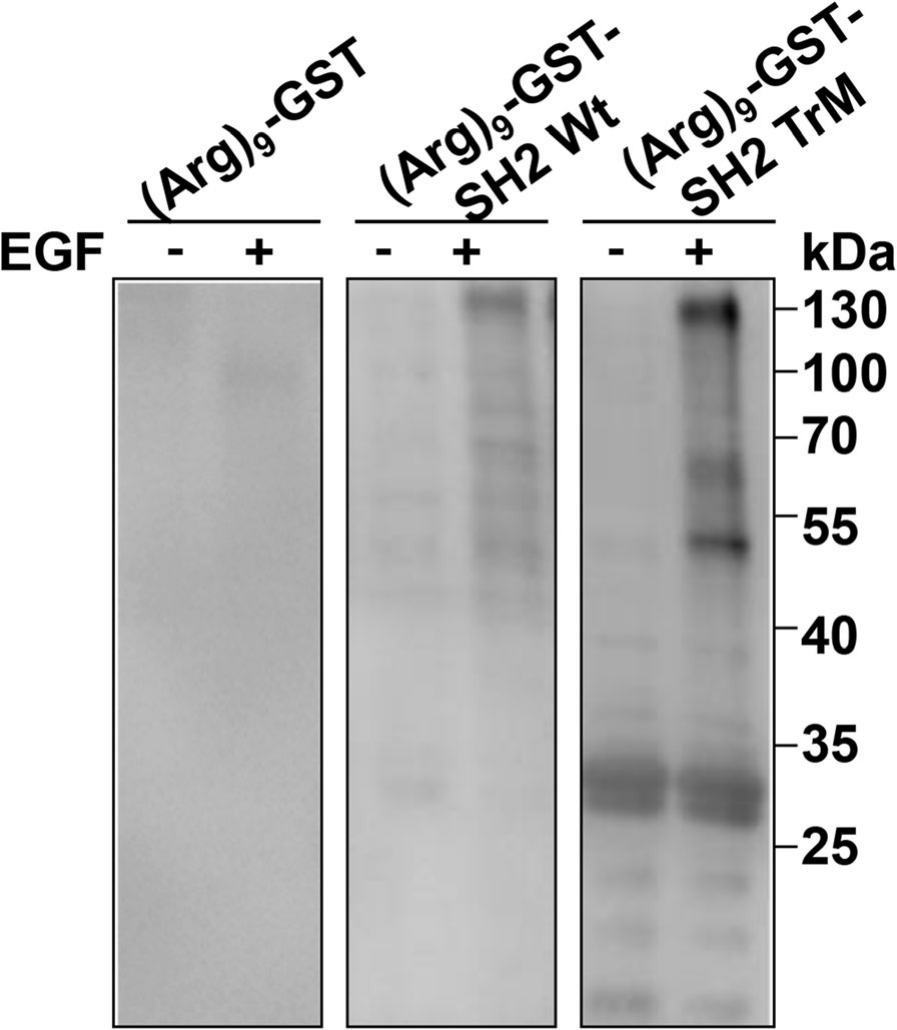
(Arg)_9_-GST SH2 TrM can effectively bind with pY proteins in B16F10 cells. The whole cell lysates of B16F10 cells with or without EGF treatment were incubated with (Arg)_9_-GST, (Arg)_9_-GST SH2 Wt or (Arg)_9_-GST SH2 TrM for 12 h at 4 °C. The pY-containing proteins were enriched, pulled down and then examined by western blot using anti-pY antibody. Data shown are representative of three independent experiments

To test whether (Arg)_9_-GST SH2 TrM could effectively bind with pY-containing proteins, the mouse melanoma B16F10 cells were subjected to serum-free medium for 16 h, and with or without the stimulation of EGF (100 ng/ml), and then treated with sodium pervanadate prior to collection. The whole cell lysates were incubated with (Arg)_9_-GST, (Arg)_9_-GST SH2 Wt or (Arg)_9_-GST SH2 TrM (0.2 mg protein each reaction) for 12 h at 4 °C, and the pY-containing proteins were enriched and pulled down by an appropriate amount of glutathione agarose. The results showed that almost no protein bands were seen in (Arg)_9_-GST control, while pY-containing proteins from (Arg)_9_-GST SH2 TrM group were more obvious than those from (Arg)_9_-GST SH2 Wt, and EGF can promote the phosphorylation level of protein tyrosine residues (Fig. 1). Levels of pY proteins in human melanoma cell A375 and its cisplatin (DDP)-resistant subline A375/DDP cells were shown in Additional file 4: Figure S2.

As SH2 domain can specifically recognize and bind to pY residues, SH2-containing proteins play a vital role in the pY-based signaling transduction [21]. (Arg)_9_-GST SH2 TrM exhibits strong binding ability with pY proteins in cells, therefore, it may competitively displace the natural SH2-containing proteins and block a number of pY-based signaling pathways in cells.

### (Arg)_9_-GST SH2 TrM could efficiently penetrate into melanoma cells

**Fig. 2:**
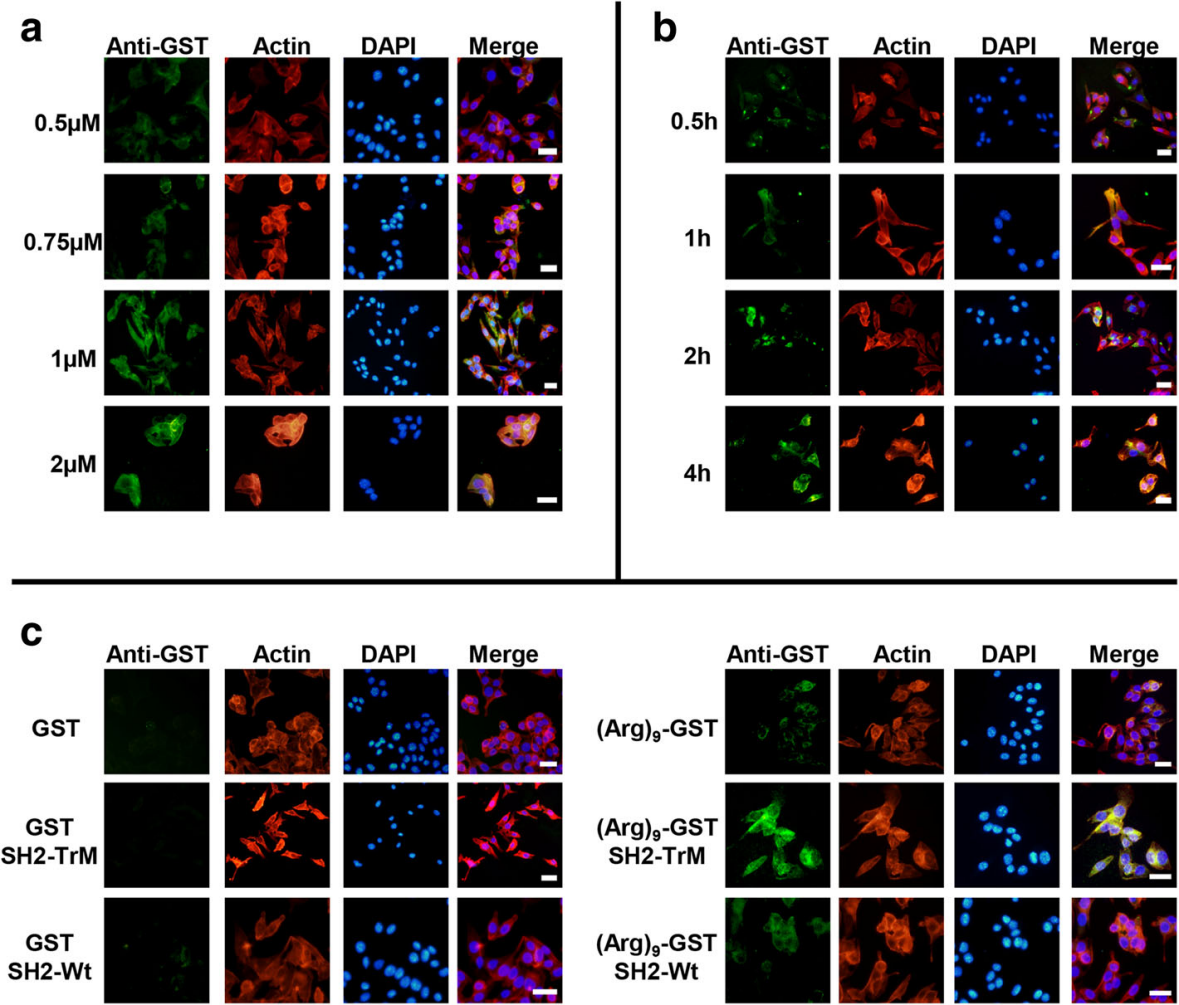
(Arg)_9_-GST SH2 TrM could efficiently enter into B16F10 cells. Effects of (Arg)9-GST SH2 TrM translocating into B16F10 cells at different concentrations (0.5, 0.75,1 and 2 μM) (**a**) and various time (0.5 h,1 h,2 h and 4 h) (**b**). **c** Distinct penetration ability of GST, GST SH2 Wt, GST SH2 TrM, (Arg)_9_-GST, (Arg)_9_-GST SH2 Wt and (Arg)_9_-GST SH2 TrM proteins. Cells were stained with anti-GST antibody followed by goat-anti-rabbit FITC secondary antibody. Actin was stained with Rhodamine-phallodin (Red) and nucleus with DAPI (Blue). Scale bar: 20 μm. All images shown are representative of at least three independent experiments

To identify the transmembrane ability of (Arg)_9_-GST SH2 TrM, B16F10 cells were incubated with proteins at different concentrations (0.5, 0.75,1 and 2 μM) for 2 h. The results showed that (Arg)_9_-GST SH2 TrM could enter cells efficiently, and 1 μM was an optimal concentration (Fig. 2a). Cells were incubated with 1 μM protein at different time (0.5 h,1 h,2 h and 4 h), and 2 h was found to be the appropriate time (Fig. 2b). As shown in Fig. 2c, (Arg)_9_-fused proteins could translocate into cells, whereas those without nona-arginine failed to do so (left panel). Furthermore, the green fluorescence signal of (Arg)_9_-GST or (Arg)_9_-GST SH2 Wt was observed to be weaker than that of (Arg)_9_-GST-SH2 TrM (right panel). (Arg)_9_-GST-SH2 TrM protein could be enriched in the cells via tightly binding with pY-containing protein complexes, so strong green fluorescence signal of (Arg)_9_-GST-SH2 TrM was observed. In contrast, (Arg)_9_-GST in the cytoplasm couldn’t bind with pY-containing protein complexes for the lacking of SH2 TrM domain, and the binding of (Arg)_9_-GST SH2 Wt to pY-containing protein complexes was moderate, thus their green fluorescence signal tended to be weak in the cells.

### (Arg)_9_-GST SH2 TrM attenuated proliferation of melanoma cells

**Fig. 3:**
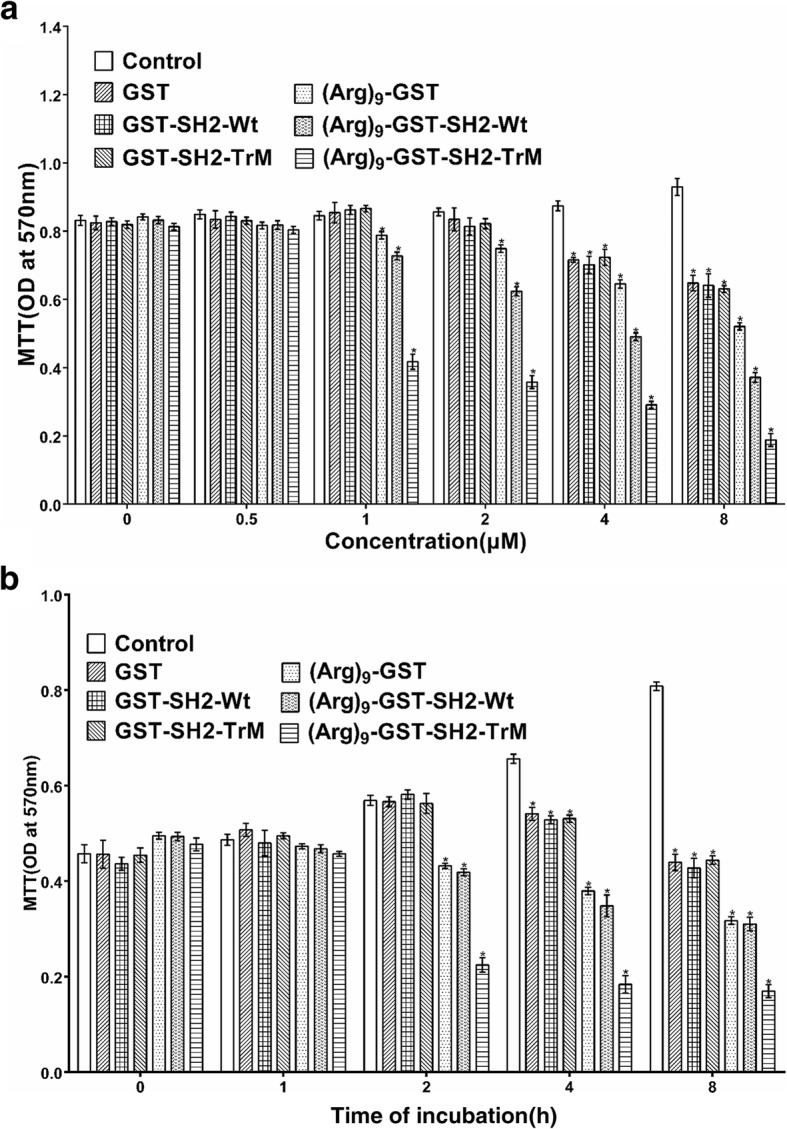
Effects of GST, GST SH2 Wt, GST SH2 TrM, (Arg)_9_-GST, (Arg)_9_-GST SH2 Wt and (Arg)_9_-GST SH2 TrM on the proliferation of B16F10 cells. Cells were treated with different GST-fused proteins or PBS (as control) at different concentrations (**a**) (0.5, 1, 2, 4 and 8 μM) for various time (**b**) (1,2,4 and 8 h) and cell viability was measured by MTT assay (*n* = 3, *P < 0.05)

**Fig. 4:**
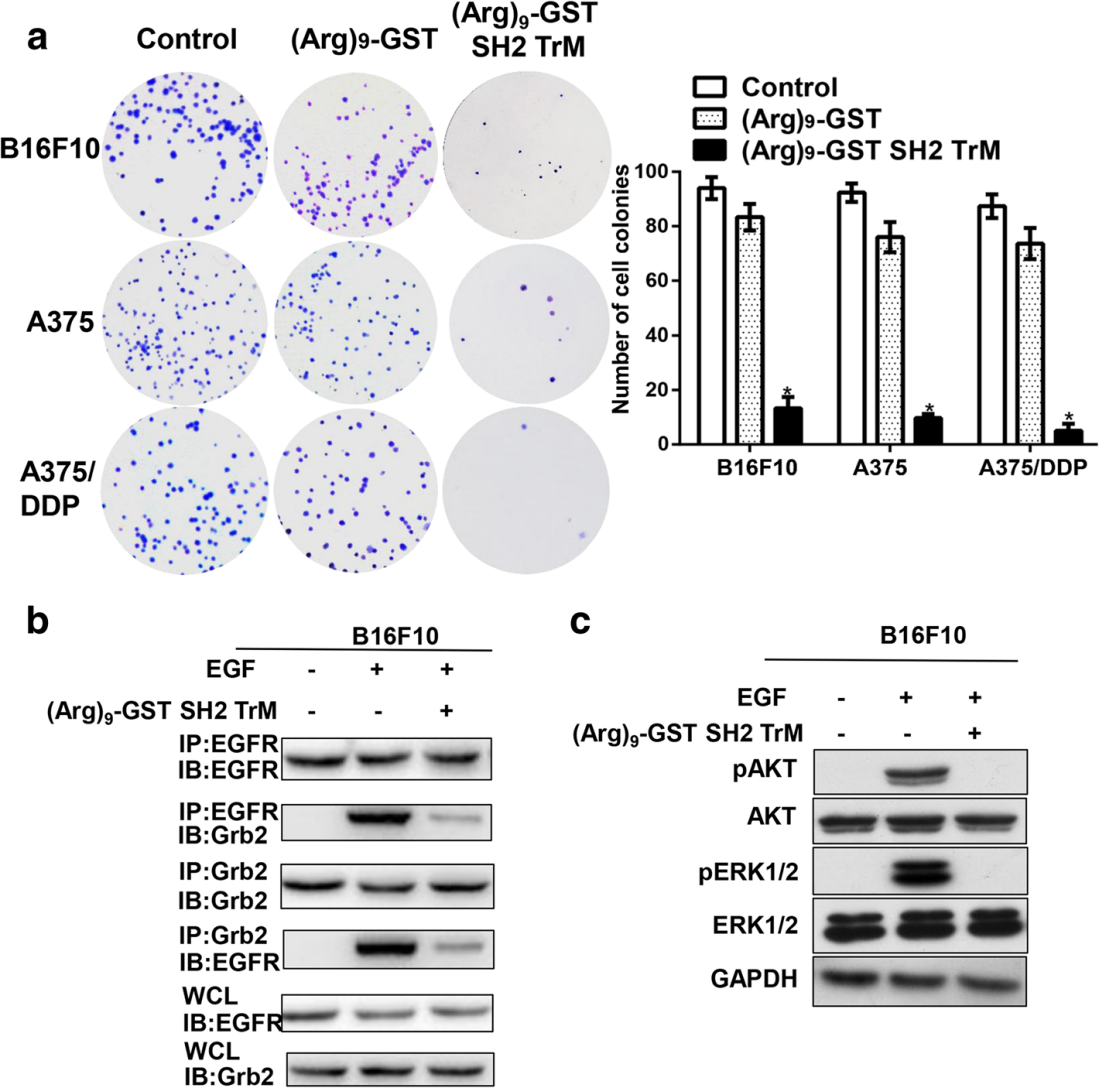
Proliferation capacity of melanoma cells was suppressed by (Arg)_9_-GST SH2 TrM. **a** Representative images of colony formation assay showing colonies formed by B16F10 cells incubated with (Arg)_9_-GST SH2 TrM or (Arg)_9_-GST (*n* = 3, *P < 0.05). **b** (Arg)_9_-GST SH2 TrM treatment blocked the EGFR-Grb2 signaling of B16F10 cells. IP, immunoprecipitation; IB, immunoblot. WCL, whole cell lysates. **c** (Arg)_9_-GST SH2 TrM treatment reduced the phosphorylation of ERK and AKT of B16F10 cells. Data shown are representative of three independent experiments

To investigate the ability of (Arg)_9_-GST SH2 TrM to inhibit the proliferation of B16F10 cells, MTT assay was conducted. After the fusion proteins, including GST, GST SH2 Wt, GST SH2 TrM, (Arg)_9_-GST, (Arg)_9_-GST SH2 Wt and (Arg)_9_-GST SH2 TrM, were used at varying concentrations of 0.5, 1, 2, 4 and 8 μM, or different treatment time of 1,2,4 and 8 h, 1 μM for 2 h was the optimal choice to attenuate the proliferation of B16F10 cells (Fig. 3a and b). The results were verified to be similar for A375 cells (Additional file 5: Figure S3) and A375/DDP cells (Additional file 6: Figure S4). To further confirm the reduction in cellular viability, colony formation assay was performed. Compared with the control group, the clonogenic ability of melanoma cells was drastically decreased after incubated with 1 μM (Arg)_9_-GST SH2 TrM (Fig. 4a). These data provided evidence that (Arg)_9_-GST SH2 TrM restrained proliferation of melanoma cells in vitro.

Targeting tyrosine kinases that can drive tumorigenesis is an effective strategy in molecular targeted therapy [22, 23]. EGFR family members are associated with many epithelial cancers [24, 25]. Due to its strong binding ability with pY-containing proteins, (Arg)_9_-GST SH2 TrM may decrease the tyrosine phosphorylation level of proteins derived from EGFR and act as an inhibitor of EGFR signaling and EGF-dependent cell growth. Moreover, Grb2, a critical adaptor protein in EGFR signaling [26], was found to bind less to EGFR after cells treated with (Arg)_9_-GST SH2 TrM (Fig. 4b). This indicated that (Arg)_9_-GST SH2 TrM effectively inhibited the EGF-dependent pathways, likely by competing for binding to the pY sites on the EGFR. Both MAPK/ERK and PI3K/AKT pathways are critical downstream pathways in the proliferation of cancer cells [27–29]. (Arg)_9_-GST SH2 TrM drastically reduced the phosphorylation of ERK and AKT(Fig. 4c), which are the downstream kinases of the EGFR signaling pathways [30]. The results of A375 and A375/DDP cells were similar to B16F10 cells, and (Arg)_9_-GST SH2 TrM reduced the phosphorylation of ERK and AKT (Additional file 7: Figure S5a). These results proved that (Arg)_9_-GST SH2 TrM blocked the EGFR signal pathway and the downstream PI3K/AKT and MAPK/ERK signaling pathways in the proliferation of melanoma cells.

### (Arg)_9_-GST SH2 TrM inhibited the migration of melanoma cells

**Fig. 5:**
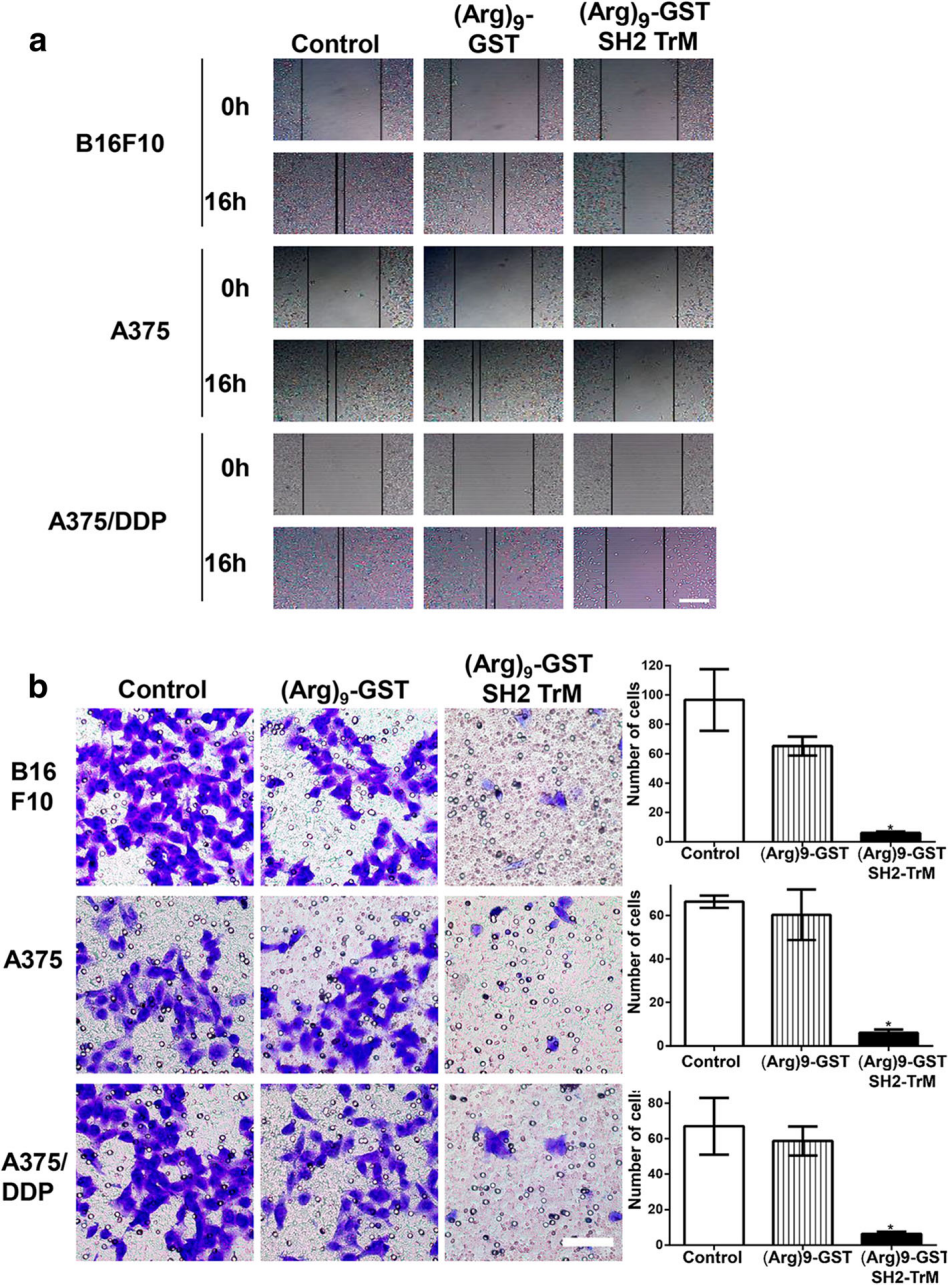
(Arg)_9_-GST SH2 TrM repressed migration of melanoma cells. **a** Wound healing assays were monitored at 0 h and 16 h for B16F10, A375 and A375/DDP cells treated with (Arg)_9_-GST SH2 TrM or (Arg)_9_-GST. Scale bar: 100 μm. **b** Transwell migration assays of B16F10, A375 and A375/DDP cells treated with (Arg)_9_-GST SH2 TrM or (Arg)_9_-GST at 1 μM for 16 h (*n* = 3, *P < 0.05). Scale bar: 20 μm. All images shown are representative of at least three independent experiments

To examine whether (Arg)_9_-GST SH2 TrM may inhibit the migration of melanoma cells, the effect of (Arg)_9_-GST SH2 TrM on migration of cells was monitored by wound healing assay. Cells were seeded in six-well plates, and cultured with complete cell medium or contained with 1 μM (Arg)_9_-GST or (Arg)_9_-GST SH2 TrM after stimulated with EGF (100 ng/ml). Melanoma cells treated with (Arg)_9_-GST SH2 TrM had limited ability of migration after scratched for 16 h (Fig. 5a). Furthermore, Transwell system was used to assess the ability of migration. Cell numbers of the group that treated with (Arg)_9_-GST SH2 TrM for 16 h were drastically less than those of the control group (Fig. 5b). These data indicated that the migration of melanoma cells was inhibited by (Arg)_9_-GST SH2 TrM.

### (Arg)_9_-GST SH2 TrM induced apoptosis of B16F10 cells

**Fig. 6:**
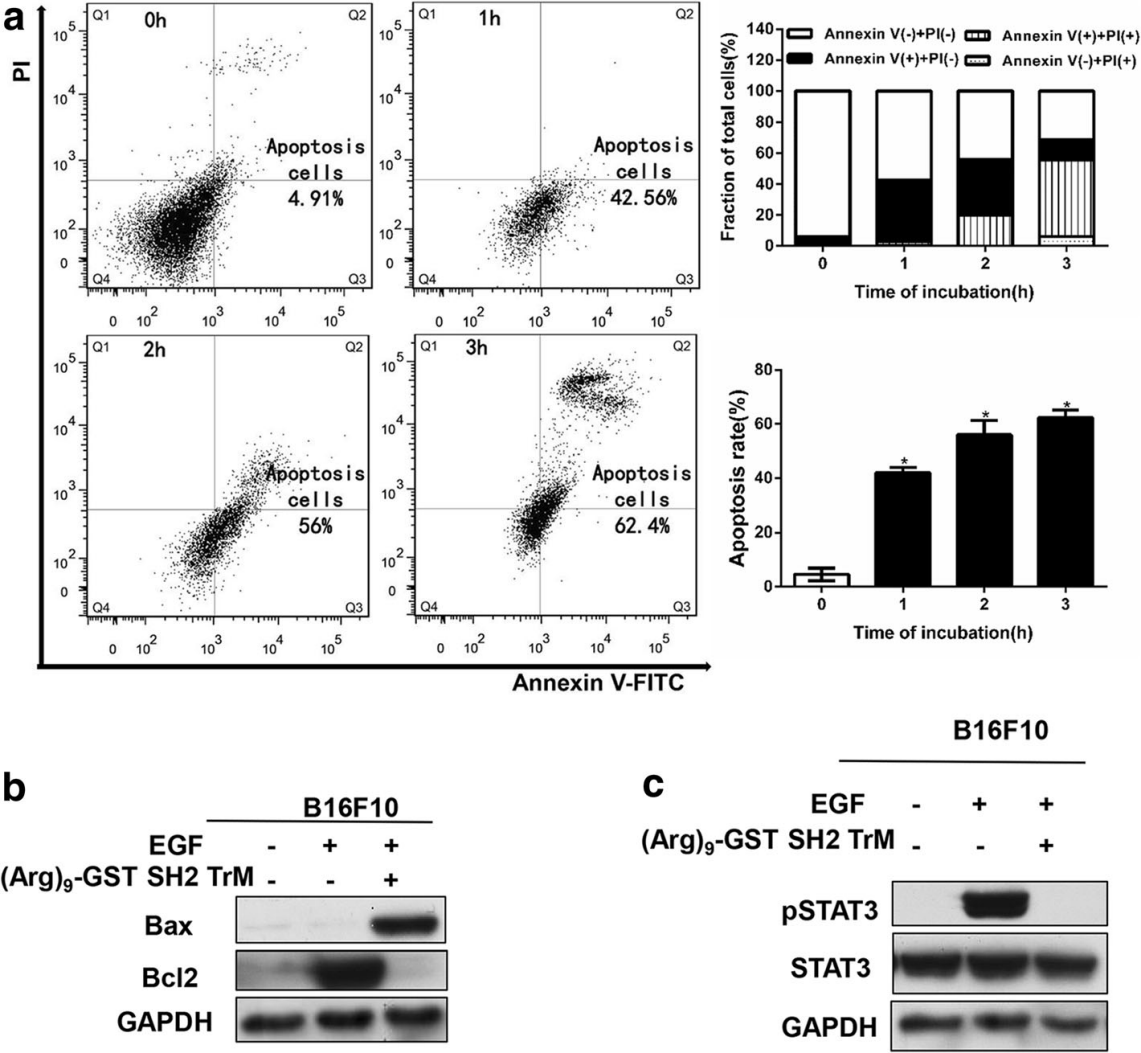
(Arg)_9_-GST SH2 TrM induced apoptotic death of B16F10 cells. **a** Flow cytometry measurement of cell apoptosis induced by (Arg)_9_-GST SH2 TrM at different time of incubation (*n* = 3, *P < 0.05). Q1: necrotic cells, Q2: late apoptotic cells; Q3: early phase apoptotic cells; Q4: normal cells. **b** (Arg)_9_-GST SH2 TrM treatment regulated the expression of Bax and Bcl-2. **c** Level of pSTAT3 and STAT3 was examined by Western Blot. GAPDH was used as a loading control. All cells were incubated with (Arg)_9_-GST SH2 TrM at 1 μM for 2 h before collected. Data shown are representative of three independent experiments

In the previous trials, the phenomenon of cell death after treated with (Arg)_9_-GST SH2 TrM appeared, and the number of dead cells increased over time. To investigate whether the cell death was induced by apoptosis, B16F10 cells were treated with (Arg)_9_-GST SH2 TrM at 1 μM for different time intervals (1 h,2 h and 3 h). The apoptotic cell fractions of B16F10 cells that received different treatment time are shown in Fig. 6a. The results showed that the number of apoptotic cells (Q2 + Q3, Q2 and Q3 represented cells at phases of late and early of apoptosis, respectively) both significantly increased as the time of incubation increasing from 0 h to 3 h. And number of necrotic cells(Q1) increased over time. As revealed by flow cytometry analysis, the percentage of apoptosis cells reached 42.56, 56 and 62.4% for 1 h, 2 h or 3 h group respectively, which is 10-fold higher than the 0 h group (Fig. 6a). These results suggested that (Arg)_9_-GST SH2 TrM facilitated cell apoptosis, which was the main cause of in vitro cell death after (Arg)_9_-GST SH2 TrM incubation.

It is known that Bax and Bcl2 play a pivotal role in the regulation process of apoptosis [31, 32]. The results showed that Bax was increased and Bcl-2 was decreased after treating with (Arg)_9_-GST SH2 TrM, enhancing that (Arg)_9_-GST SH2 TrM could promote apoptosis in B16F10 cells (Fig. 6b). JAK/STAT signaling pathway is involved in cell cycle progression and apoptosis [33, 34]. STAT3 was detected to understand the mechanism of apoptosis caused by (Arg)_9_-GST SH2 TrM. The pSTAT3 level was declined after treated with (Arg)_9_-GST SH2 TrM (Fig. 6c). As shown in Additional file 7: Figure S5b, the phosphorylation of STAT3 also decreased in the A375 and A375/DDP cells. These data revealed that (Arg)_9_-GST SH2 TrM might lead to melanoma cell death progression via blocking JAK/STAT signal pathway.

### (Arg)_9_-GST SH2 TrM suppressed the growth of B16F10 cells on tumor-bearing mice

**Fig. 7:**
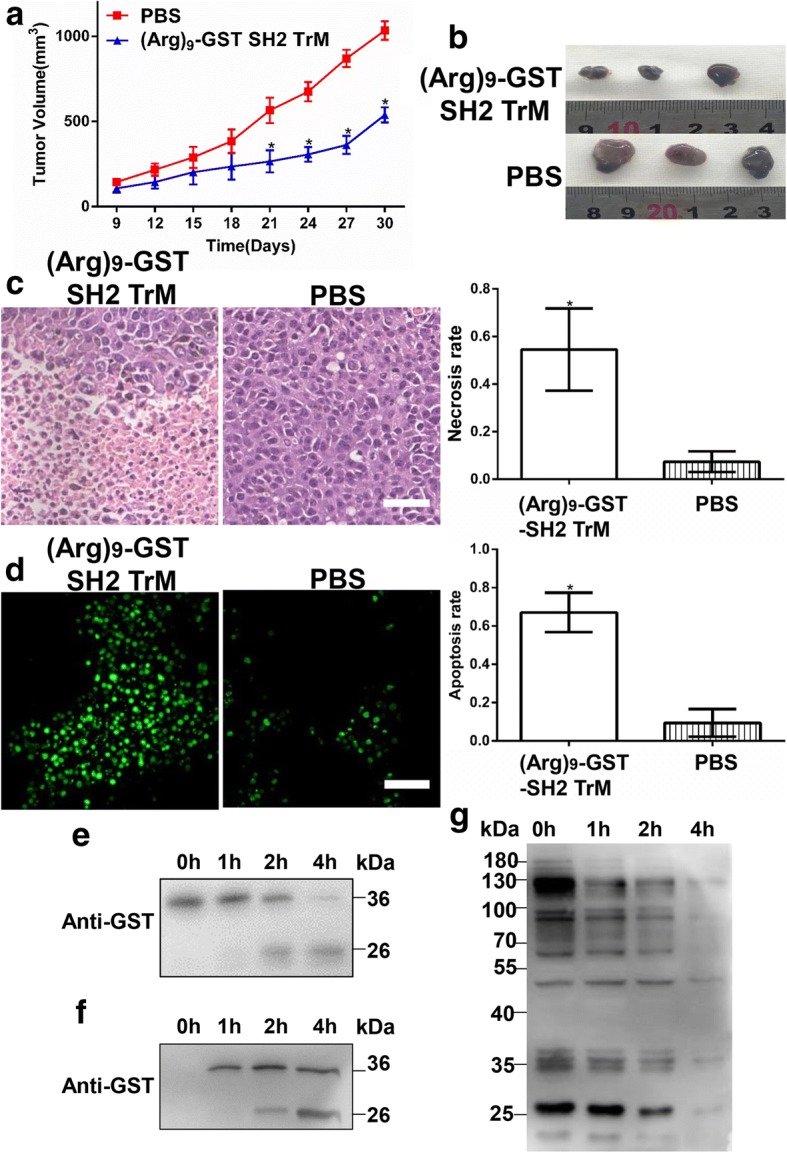
(Arg)_9_-GST SH2 TrM resulted in apoptosis and inhibited proliferation of B16F10 cells in vivo. **a** Effect of (Arg)_9_-GST SH2 TrM on the growth of B16F10 tumors. The changes of tumor volumes were shown between (Arg)_9_-GST SH2 TrM and PBS control (*n* = 3, *P < 0.05). **b** Tumors were resected after 30 days of treatment. Tumor volumes from (Arg)_9_-GST SH2 TrM group were smaller than the PBS group. **c** HE staining of xenograft tumors from mice injected with (Arg)_9_-GST SH2 TrM (left) or PBS (right). (Arg)_9_-GST SH2 TrM resulted in high level of necrosis lesions in tumors. Scale bar: 50 μm. **d** TUNEL assay for B16F10 tumors after treated with or without (Arg)_9_-GST SH2 TrM. The apoptotic nuclei were stained with a fluorescent marker (green). Scale bar: 50 μm. **e** Degradation of (Arg)_9_-GST SH2 TrM in normal mouse serum was evaluated by Western Blot with anti-GST antibody. **f** and **g** Tumors were resected and lysated at different time points after the injection of (Arg)_9_-GST SH2 TrM protein. The level of GST-fused proteins in tumor tissue was examined by Western Blot with anti-GST antibody **(f**). The phosphorylation level of tyrosine proteins in tumor tissue was checked by Western Blot with anti-pY antibody. All images shown are representative of at least three independent experiments

To further confirm the role of (Arg)_9_-GST SH2 TrM in vivo, mice were injected subcutaneously with 1 × 10^6^ B16F10 cells on the right flanks. Since the 9th day, mice were injected with (Arg)_9_-GST SH2 TrM or PBS through tail vein twice a day (10 mg/kg). Tumor volumes of (Arg)_9_-GST SH2 TrM group were found to be much smaller than those of the PBS group. The results demonstrated that (Arg)_9_-GST SH2 TrM resulted in pronounced regression of B16F10 tumors (Fig. 7a and b). HE staining was utilized to observe the changes of tumor cell morphology. The images illustrated (Arg)_9_-GST SH2 TrM resulted in high level of necrosis lesions in tumors (Fig. 7c). The sections of tumors were assayed for apoptosis analysis using a TUNEL kit that labels apoptotic nuclei with a green fluorescent marker. The incidence of apoptosis in (Arg)_9_-GST SH2 TrM-treated group was significantly higher than PBS group (Fig. 7d). These data demonstrated that (Arg)_9_-GST SH2 TrM could be an effective therapy to melanoma and induce apoptosis of B16F10 melanoma in vivo. The ability of (Arg)_9_-SH2 superbinder to reduce the tumor burden in xenograft mice suggested that (Arg)_9_-SH2 superbinder represented a novel strategy for the treatment of melanoma.

Undoubtedly, (Arg)_9_-GST-SH2 TrM protein must subject to the proteolysis in the bloodstream. To investigate its stability in blood, (Arg)_9_-GST-SH2 TrM protein was added into the dish with normal mouse serum (Abbkine, China) and the dish was gently shaken under 37 °C to mimic the condition of bloodstream. As shown in Fig. 7e, degradation of (Arg)_9_-GST SH2 TrM in the serum was checked by Western Blot with anti-GST antibody, and the fusion protein almost degraded at the time point of 4 h. At the same time, the stability of (Arg)_9_-GST-SH2 TrM protein in the lysated tumor tissue from B16F10 Xenograft mice was checked. The total level of GST-fused protein gradually increased from 1 h to 4 h, but (Arg)_9_-GST-SH2 TrM protein was observed to obviously degrade at the time point of 4 h (Fig. 7f). Furthermore, the phosphorylation level of tyrosine proteins in the lysated tumor tissue was examined by Western Blot with anti-pY antibody. The phosphorylation level of tyrosine proteins in the lysated tumor tissue from B16F10 Xenograft mice drastically decreased at 4 h after the injection of (Arg)_9_-GST-SH2 TrM (Fig. 7g), which indicated that (Arg)_9_-GST-SH2 TrM protein significantly blocked the pY-based signaling pathways by the strong binding with pY proteins in the tumor cells. All above data demonstrated that (Arg)_9_-GST-SH2 TrM protein exhibited excellent anti-tumor functions in spite of the proteolysis in vivo.

## Discussion

Patients, with BRAF (V600) in 40–60% of melanomas, can be successfully cured with selective inhibitors, bringing about significant prolongation of progression free survival and overall survival [35, 36]. Although BRAF inhibitors are effective treatment methods, drug resistance is inevitable, and high drug prices increase the burden on patients [37–39]. Meanwhile, the remaining 50–60% of patients are BRAF wild type and they do not benefit from treatment with BRAF inhibitors. A number of other mutations are present in these patients, such as NRAS, MEK1/2 and atypical (non-V600) BRAF mutations [8]. Treatment of this subset of patients without a BRAFV600 mutation is a challenging problem.

EGF, an extracellular signal factor, can activate downstream Ras, by binding with the EGFR, and then phosphorylate Raf, decreasing Ras-GTPase activity. The EGF-dependent signal pathways are closely related with tumorigenesis [40]. BRAF, an important member of Raf family, is the downstream effector of RAS. Protein tyrosine phosphorylation plays an essential role in the development and progression of cancer. Aberrant tyrosine phosphorylation is associated with cancer [41, 42]; Carcinogenic cells tend to exhibit abnormal high levels of phosphorylated tyrosine [43, 44]. Previous studies have proved that the inactivation of MAPK/ERK signaling pathways can regulate migration of HEK293T cells [16]. The tight combination of SH2 superbinder and EGFR could block the activation of downstream signaling pathways [16]. Similarly, our results substantiated that levels of pAKT, pERK1/2 and pSTAT3 were reduced by (Arg)_9_-SH2 superbinder via blocking the EGFR signaling pathway. Remarkably, the effects of (Arg)_9_-SH2 superbinder on pathways described above were involved in the regulation of cell apoptosis. There are a large number of members in Bcl-2 family involved in regulation of apoptosis, such as Bcl-2, Bcl-X1, Bcl-w, Bax, Bad, Bak, Bcl-xs, Mc1–1 et al. Bcl-2 and Bax, two most popular members of this family, were chosen to be examined in this study. The results indicated pro-apoptotic protein Bax increased and anti-apoptosis protein Bcl-2 declined in B16F10 cells after (Arg)_9_-SH2 superbinder incubation. Of course, it needs further investigations to check whether other members in Bcl-2 family mediate the process of (Arg)_9_-SH2 superbinder-induced apoptosis in future.

In addition, the powerful vector (Arg)_9_, for the intracellular delivery of conjugated large molecules, was applied efficaciously to translocate across the cell membrane and transport SH2 superbinder into the cytoplasm or nucleus of B16F10 cells. The results showed that fluorescence intensity of cells incubated with (Arg)_9_-GST SH2 TrM was higher than that of (Arg)_9_-GST because of the strong affinity between (Arg)_9_-GST SH2 TrM and pY-containing proteins in cells. Indeed, cell penetrating peptides inevitably have some toxicity effect on the cells. The MTT data in our work showed that (Arg)_9_-GST protein exhibited remarkably low toxicity, which is consistent with previous reports [45, 46]. In brief, (Arg)_9_ overcame the challenge of transporting therapeutic agents across cell membrane which showed a great value of application in biomedical system. Furthermore, we isolated the peripheral blood mononuclear cells(PBMCs) from mice and evaluated the penetration and toxicity effect of (Arg)_9_ fused proteins on PBMCs. As shown in Additional file 8: Figure S6a, the level of pY was very low in PBMCs as the non-cancerous cells. (Arg)_9_-GST-SH2 TrM protein could enter into PBMCs, but the green signal was very weak (Additional file 8: Figure S6b). Indeed, (Arg)_9_-GST-SH2 TrM protein caused some toxicity effect on PBMCs according to the CCK-8 data (Additional file 9: Figure S7), but it was moderate as (Arg)_9_-GST-SH2 TrM protein specifically recognizing and binding to pY residues, whereas the level of pY residues was verified to be very low in PBMCs.

These findings suggested that (Arg)_9_-SH2 superbinder could play a significant role in the progression of cell proliferation, migration and apoptosis. Importantly, these data implied that (Arg)_9_-SH2 superbinder, targeting multiple pY proteins, might be a useful anticancer therapy to address the difficulties of resistance and provide a novel effective way for melanoma.

## Conclusions

In summary, it is the first time to combine (Arg)_9_ with SH2 superbinder to form a drug delivery system. The findings indicated (Arg)_9_-SH2 superbinder had potent antitumor ability by targeting various pY proteins and blocking diverse pY-based signaling pathways. The results verified that (Arg)_9_-SH2 superbinder could suppress proliferation, migration, and induce apoptosis of melanoma cells via regulating the activity of PI3K/AKT, MAPK/ERK and JAK/STAT signal pathways. (Arg)_9_-SH2 superbinder had the potential to act as a promising therapy for melanoma and displayed a prospective value of application.

## Additional files

Additional file 1: **Table S1.** The amino acid sequences of constructs (N-C). (PDF 323 kb)

Additional file 2: **Table S2.** Sequence of primers. (PDF 69 kb)

Additional file 3: **Figure S1.** Construction, expression and purification of GST fusion proteins. (a) DNA gel electrophoresis image showing the molecular weights of the DNA fragment of SH2 TrM and pGEX-4 T3-(Arg)9-SH2 TrM plasmid. (b) SDS-PAGE Coomassie blue-staining image displaying the expression and purification of (Arg)9-GST, (Arg)9-GST SH2 Wt and (Arg)9-GST SH2 TrM in E.coli. Data shown are representative of three independent experiments. (PPTX 163 kb)

Additional file 4: **Figure S2.** (Arg)9-GST SH2 TrM could effectively capture diverse pY proteins in A375 and A375/DDP cells. Levels of pY proteins from A375(a) and A375/DDP(b) cells stimulated with or without EGF (100 ng/ml) were assessed with Anti-pY antibody. The whole cell lysates were incubated with (Arg)9-GST, (Arg)9-GST SH2 Wt or (Arg)9-GST SH2 TrM for 12 h at 4 °C and purified by an appropriate amount of glutathione agarose. Data shown are representative of three independent experiments. (PPTX 107 kb)

Additional file 5: **Figure S3.** (Arg)9-GST SH2 TrM inhibited the proliferation of A375 cells. Effects of GST, GST SH2 Wt, GST SH2 TrM, (Arg)9-GST, (Arg)9-GST SH2 Wt and (Arg)9-GST SH2 TrM on the proliferation of A375 cells. Cells were treated with different GST-fused proteins at different concentrations (a) (0.5,1, 2, 4 and 8 μM) for various time (b) (1,2,4 and 8 h) and cell viability was measured by MTT assay (*n* = 3, *P < 0.05). (PPTX 366 kb)

Additional file 6: **Figure S4.** (Arg)9-GST SH2 TrM inhibited the proliferation of A375/DDP cells. Effects of GST, GST SH2 Wt, GST SH2 TrM, (Arg)9-GST, (Arg)9-GST SH2 Wt and (Arg)9-GST SH2 TrM on the proliferation of A375/DDP cells. Cells were treated with different GST-fused proteins at different concentrations (a) (0.5, 1, 2, 4 and 8 μM) for various time (b) (1,2,4 and 8 h) and cell viability was measured by MTT assay (*n* = 3, *P < 0.05). (PPTX 373 kb)

Additional file 7: **Figure S5.** Effects of (Arg)9-GST SH2 TrM on JAK/STAT, MAPK/ERK and PI3K/AKT pathways of A375 and A375/DDP cells. Variant levels of phosphorylated and total ERK, AKT (a) and STAT3 (b) from A375 and A375/DDP cells were detected by Western Blot. Cells were treated with or without EGF and (Arg)9-GST SH2 TrM before harvesting. Data shown are representative of three independent experiments. (PPTX 223 kb)

Additional file 8: **Figure S6.** The penetration and toxicity effects of (Arg)9-GST SH2 TrM on PBMCs. (a) The level of pY proteins was very low in PBMCs compared with B16F10 cells. (b) (Arg)9-GST-SH2 TrM protein could enter into PBMCs, but could not efficiently be enriched by pY proteins in these non-cancerous cells, thus the green signal was observed to be weak. All images shown are representative of at least three independent experiments. (PPTX 329 kb)

Additional file 9: **Figure S7.** The toxicity effect of (Arg)9-GST SH2 TrM on PBMCs. (Arg)9-GST-SH2 TrM protein resulted in some toxicity effect on PBMCs according to the CCK-8 data, but the effect was moderate as (Arg)9-GST-SH2 TrM protein only specifically recognizing and binding to pY residue, which is low abundance in PBMCs. Cells were treated with different GST-fused proteins at different concentrations (a) (0.5, 1, 2, 4 and 8 μM) for various time (b) (1,2,4 and 8 h) and cell viability was measured by CCK-8 assay (*n* = 3, *P < 0.05). (PPTX 352 kb)

## Abbreviations

(Arg)_9_: Nine arginines
(Arg)_9_-SH2 superbinder: (Arg)_9_-GST-Src SH2 TrM
CPPs: Cell penetrating peptides
pY: Phosphorylated tyrosine
SH2 TrM: SH2 superbinder, the Src Homology 2 (SH2) domain with the triple-mutant Thr8Val/Cys10Ala/Lys15Leu
Wt: wild type
