# Supplementary material for: (Arg)9-SH2 superbinder: a novel promising anticancer therapy to melanoma by blocking phosphotyrosine signaling
Source: J Exp Clin Cancer Res. 2018 Jul 5;37:138. doi: 10.1186/s13046-018-0812-5 (PMC6034221; doi:10.1186/s13046-018-0812-5)
Supplement: Supplementary file 1 — Table S1. The amino acid sequences of constructs (N-C). (PDF 323 kb) [file 13046_2018_812_MOESM1_ESM.pdf]

## Table S1 The amino acid sequences of constructs (N-C).

| | |
|---|---|
| Src SH2 Wt | WYFGKITRRESERLLLNAENPRGTFLVRESETTKGAYCLSVSDFDNAKGLNVKHYKIRKLDSGGFYITSRTQFNSLQQLVAYYSKHADGLCH<br>RLTTVC |
| Src SH2 TrM | WYFGKITRRESERLLLNAENPRGTFLVRESET**V**KGAY**A**LSVSDFDNAKGLNVKHY**L**IRKLDSGGFYITSRTQFNSLQQLVAYYSKHADGLC<br>HRLTTVC |
| GST-Src SH2 Wt-(Arg)$_9$ | MSPILGYWKIKGLVQPTRLLLEYLEEKYEEHLYERDEGDKWRNKKFELGLEFPNLPYYIDGDVKLTQSMAIIRYIADKHNMLGGCPKERAEI<br>SMLEGAVLDIRYGVSRIAYSKDFETLKVDFLSKLPEMLKMFEDRLCHKTYLNGDHVTHPDFMLYDALDVVLYMDPMCLDAFPKLVCFKKR<br>IEAIPQIDKYLKSSKYIAWPLQGWQATFGGGDHPPKSDLVPRGSDSIQAEEWYFGKITRRESERLLLNAENPRGTFLVRESETTKGAYCLSVS<br>DFDNAKGLNVKHYKIRKLDSGGFYITSRTQFNSLQQLVAYYSKHADGLCHRLTTVCPTSKGRRRRRRRRR |
| GST-Src SH2 TrM-(Arg)$_9$ | MSPILGYWKIKGLVQPTRLLLEYLEEKYEEHLYERDEGDKWRNKKFELGLEFPNLPYYIDGDVKLTQSMAIIRYIADKHNMLGGCPKERAEI<br>SMLEGAVLDIRYGVSRIAYSKDFETLKVDFLSKLPEMLKMFEDRLCHKTYLNGDHVTHPDFMLYDALDVVLYMDPMCLDAFPKLVCFKKR<br>IEAIPQIDKYLKSSKYIAWPLQGWQATFGGGDHPPKSDLVPRGSDSIQAEEWYFGKITRRESERLLLNAENPRGTFLVRESET**V**KGAY**A**LSVS<br>DFDNAKGLNVKHY**L**IRKLDSGGFYITSRTQFNSLQQLVAYYSKHADGLCHRLTTVCPTSKGRRRRRRRRR |
| GST-(Arg)$_9$ | MSPILGYWKIKGLVQPTRLLLEYLEEKYEEHLYERDEGDKWRNKKFELGLEFPNLPYYIDGDVKLTQSMAIIRYIADKHNMLGGCPKERAEI<br>SMLEGAVLDIRYGVSRIAYSKDFETLKVDFLSKLPEMLKMFEDRLCHKTYLNGDHVTHPDFMLYDALDVVLYMDPMCLDAFPKLVCFKKR<br>IEAIPQIDKYLKSSKYIAWPLQGWQATFGGGDHPPKSDLVPRGSRRRRRRRRR |
| GST-Src SH2 Wt | MSPILGYWKIKGLVQPTRLLLEYLEEKYEEHLYERDEGDKWRNKKFELGLEFPNLPYYIDGDVKLTQSMAIIRYIADKHNMLGGCPKERAEI<br>SMLEGAVLDIRYGVSRIAYSKDFETLKVDFLSKLPEMLKMFEDRLCHKTYLNGDHVTHPDFMLYDALDVVLYMDPMCLDAFPKLVCFKKR<br>IEAIPQIDKYLKSSKYIAWPLQGWQATFGGGDHPPKSDLVPRGSDSIQAEEWYFGKITRRESERLLLNAENPRGTFLVRESETTKGAYCLSVS<br>DFDNAKGLNVKHYKIRKLDSGGFYITSRTQFNSLQQLVAYYSKHADGLCHRLTTVCPTSK |

| | |
|---|---|
| GST-Src SH2 TrM | MSPILGYWKIKGLVQPTRLLLEYLEEKYEEHLYERDEGDKWRNKKFELGLEFPNLPYYIDGDVKLTQSMAIIRYIADKHNMLGGCPKERAEISMLEGAVLDIRYGVSRIAYSKDFETLKVDFLSKLPEMLKMFEDRLCHKTYLNGDHVTHPDFMLYDALDVVLYMDPMCLDAFPKLVCFKKRIEAIPQIDKYLKSSKYIAWPLQGWQATFGGGDHPPKSDLVPRGSDSIQAEEWYFGKITRRESERLLLNAENPRGTFLVRESET**V**KGAY**A**LSVSDFDNAKGLNVKHY**L**IRKLDSGGFYITSRTQFNSLQQLVAYYSKHADGLCHRLTTVCPTSK |

Src SH2 Wt or TrM sequences are underlined with wavy line. Tripe mutant sites are marked in red and **bold**. GST sequences are underlined with straight line. Sequence of $(Arg)_9$ was underlined with dotted line. GST-(Arg)9 and GST-Src-SH2-Wt/TrM-(Arg)9 were termed as (Arg)9-GST and (Arg)9-GST-Src-SH2-Wt/TrM in the manuscript.
