# Supplementary material for: (Arg)9-SH2 superbinder: a novel promising anticancer therapy to melanoma by blocking phosphotyrosine signaling
Source: J Exp Clin Cancer Res. 2018 Jul 5;37:138. doi: 10.1186/s13046-018-0812-5 (PMC6034221; doi:10.1186/s13046-018-0812-5)
Supplement: Supplementary file 2 — Table S2. Sequence of primers. (PDF 69 kb) [file 13046_2018_812_MOESM2_ESM.pdf]

**Table S2**

## Sequences of Primers

| Plasmid | Forward Primers(5'-3') | Reverse Primers(5'-3') |
|---|---|---|
| pGEX-4T3-Src SH2 TrM-$(Arg)_9$ | ATCTGGTTCCGCGTGGATCCGACTCCATCCAGGCTGAGG | AGTCACGATGCGGCCGCTACGGCGGCGACGACGGCGGCGACGACGGCCCTTGGACGTGGGGCACAC |
| pGEX-4T3-Src SH2 Wt-$(Arg)_9$ | ATCTGGTTCCGCGTGGATCCGACTCCATCCAGGCTGAGG | AGTCACGATGCGGCCGCTACGGCGGCGACGACGGCGGCGACGACGGCCCTTGGACGTGGGGCACAC |
| pGEX-4T3-Src SH2 TrM | ATCTGGTTCCGCGTGGATCCGACTCCATCCAGGCTGAGG | AGTCACGATGCGGCCGCTCTTGGACGTGGGGCACAC |
| pGEX-4T3-Src SH2 Wt | ATCTGGTTCCGCGTGGATCCGACTCCATCCAGGCTGAGG | AGTCACGATGCGGCCGCTCTTGGACGTGGGGCACAC |
| pGEX-4T3-$(Arg)_9$ | ATCTGGTTCCGCGTGGATCCCGTCGTCGCCGC | AGTCACGATGCGGCCGCTACGGCGGCGACGAC |
