# Supplementary material for: (Arg)9-SH2 superbinder: a novel promising anticancer therapy to melanoma by blocking phosphotyrosine signaling
Source: J Exp Clin Cancer Res. 2018 Jul 5;37:138. doi: 10.1186/s13046-018-0812-5 (PMC6034221; doi:10.1186/s13046-018-0812-5)
Supplement: Supplementary file 3 — Figure S1. Construction, expression and purification of GST fusion proteins. (a) DNA gel electrophoresis image showing the molecular weights of the DNA fragment of SH2 TrM and pGEX-4 T3-(Arg)9-SH2 TrM plasmid. (b) SDS-PAGE Coomassie blue-staining image displaying the expression and purification of (Arg)9-GST, (Arg)9-GST SH2 Wt and (Arg)9-GST SH2 TrM in E.coli. Data shown are representative of three independent experiments. (PPTX 163 kb) [file 13046_2018_812_MOESM3_ESM.pptx]

## Slide 1
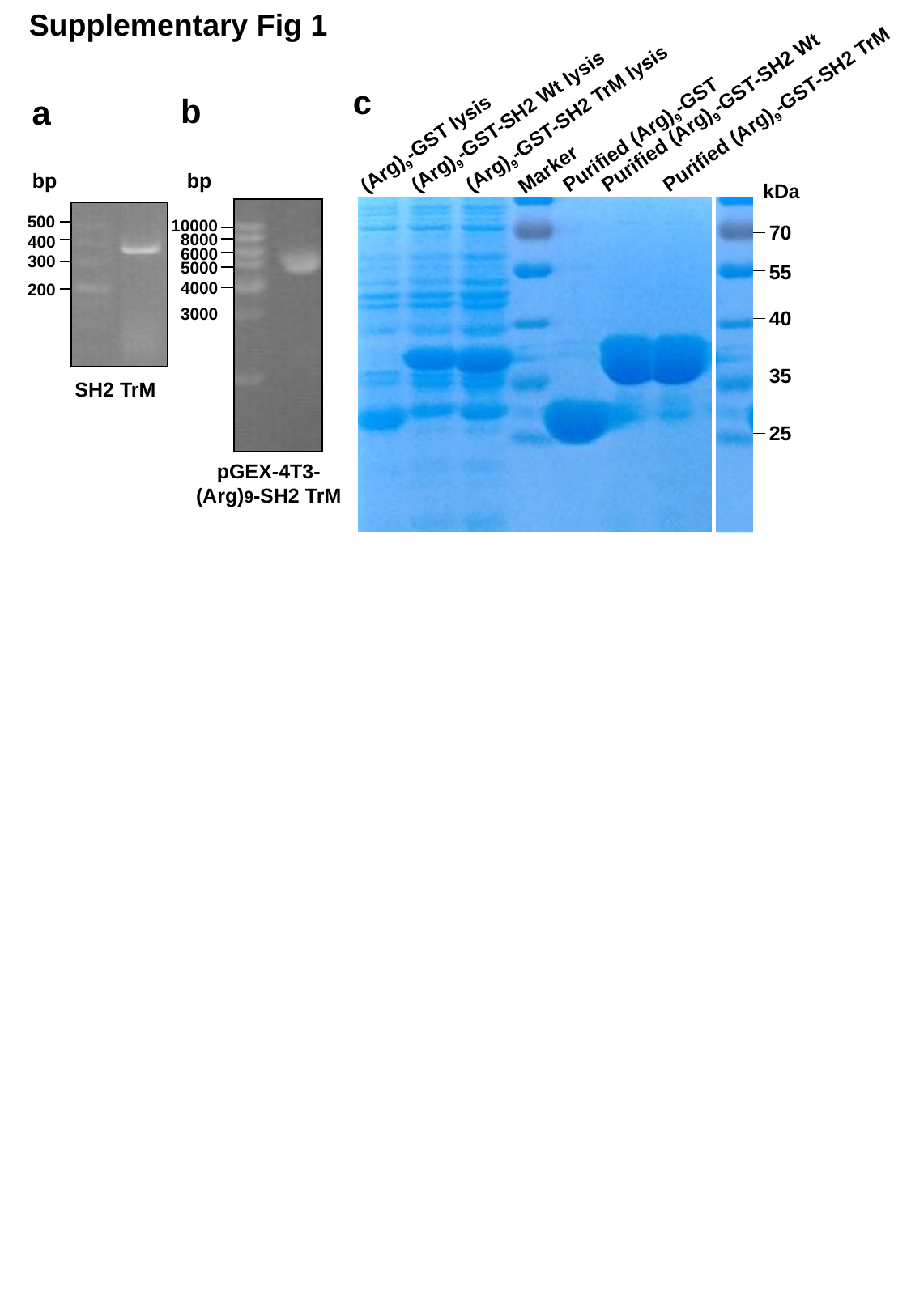

Supplementary Fig 1
c
b
a
Purified (Arg)9-GST-SH2 TrM
Purified (Arg)9-GST-SH2 Wt
(Arg)9-GST-SH2 TrM lysis
(Arg)9-GST-SH2 Wt lysis
Purified (Arg)9-GST
(Arg)9-GST lysis
Marker
bp
500
400
300
200
SH2 TrM
bp
10000
8000
6000
5000
4000
3000
pGEX-4T3-
(Arg)9-SH2 TrM
kDa
70
55
40
35
25
