# Supplementary material for: (Arg)9-SH2 superbinder: a novel promising anticancer therapy to melanoma by blocking phosphotyrosine signaling
Source: J Exp Clin Cancer Res. 2018 Jul 5;37:138. doi: 10.1186/s13046-018-0812-5 (PMC6034221; doi:10.1186/s13046-018-0812-5)
Supplement: Supplementary file 4 — Figure S2. (Arg)9-GST SH2 TrM could effectively capture diverse pY proteins in A375 and A375/DDP cells. Levels of pY proteins from A375(a) and A375/DDP(b) cells stimulated with or without EGF (100 ng/ml) were assessed with Anti-pY antibody. The whole cell lysates were incubated with (Arg)9-GST, (Arg)9-GST SH2 Wt or (Arg)9-GST SH2 TrM for 12 h at 4 °C and purified by an appropriate amount of glutathione agarose. Data shown are representative of three independent experiments. (PPTX 107 kb) [file 13046_2018_812_MOESM4_ESM.pptx]

## Slide 1
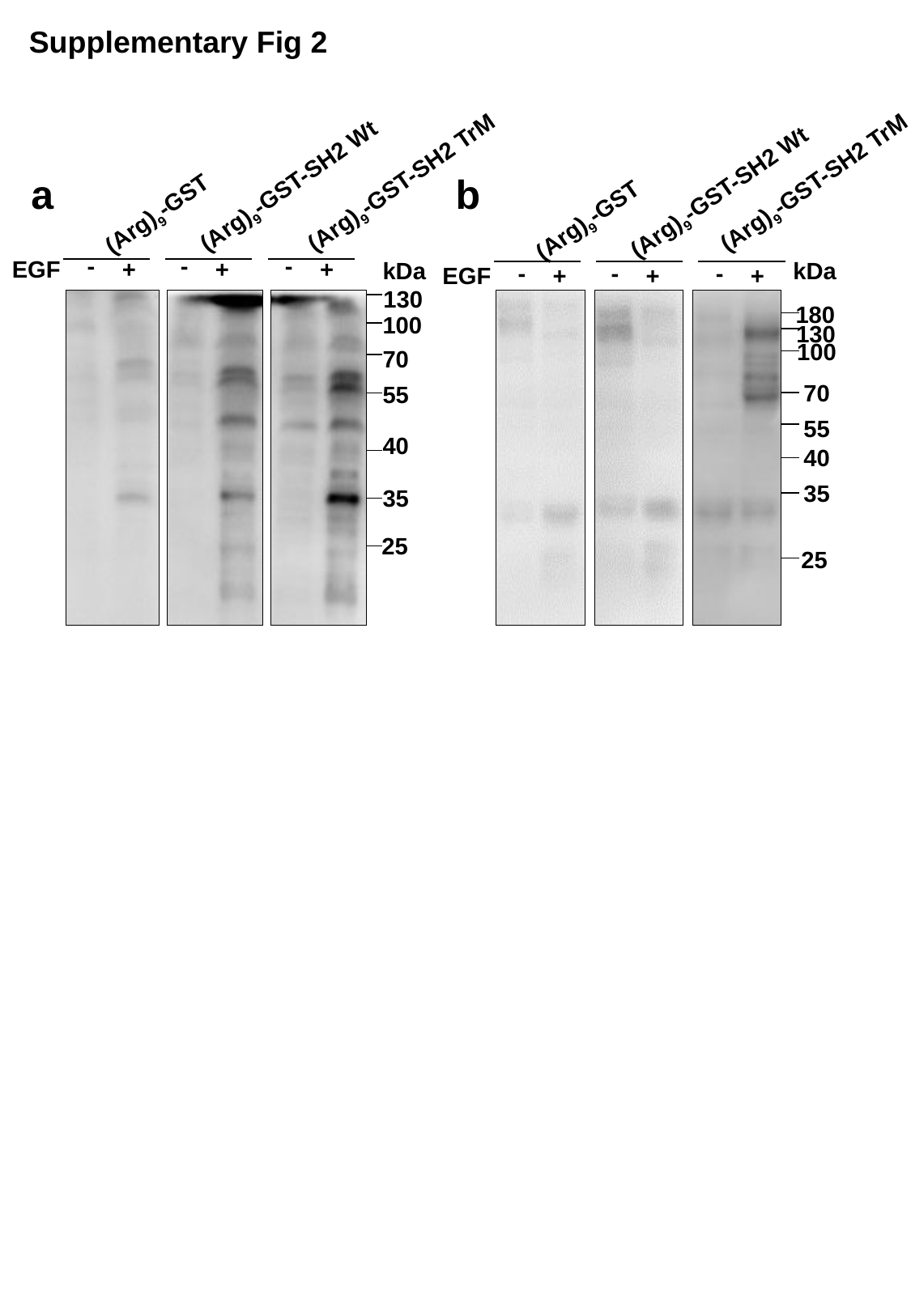

Supplementary Fig 2
a
b
 (Arg)9-GST-SH2 TrM
 (Arg)9-GST-SH2 TrM
(Arg)9-GST-SH2 Wt
(Arg)9-GST-SH2 Wt
(Arg)9-GST
(Arg)9-GST
-
-
-
EGF
+
+
+
kDa
kDa
-
-
-
EGF
+
+
+
130
180
100
130
100
70
70
55
55
40
40
35
35
25
25
