# Supplementary figures and images for: (Arg)9-SH2 superbinder: a novel promising anticancer therapy to melanoma by blocking phosphotyrosine signaling
Source: J Exp Clin Cancer Res. 2018 Jul 5;37:138. doi: 10.1186/s13046-018-0812-5 (PMC6034221; doi:10.1186/s13046-018-0812-5)

## Slide 1
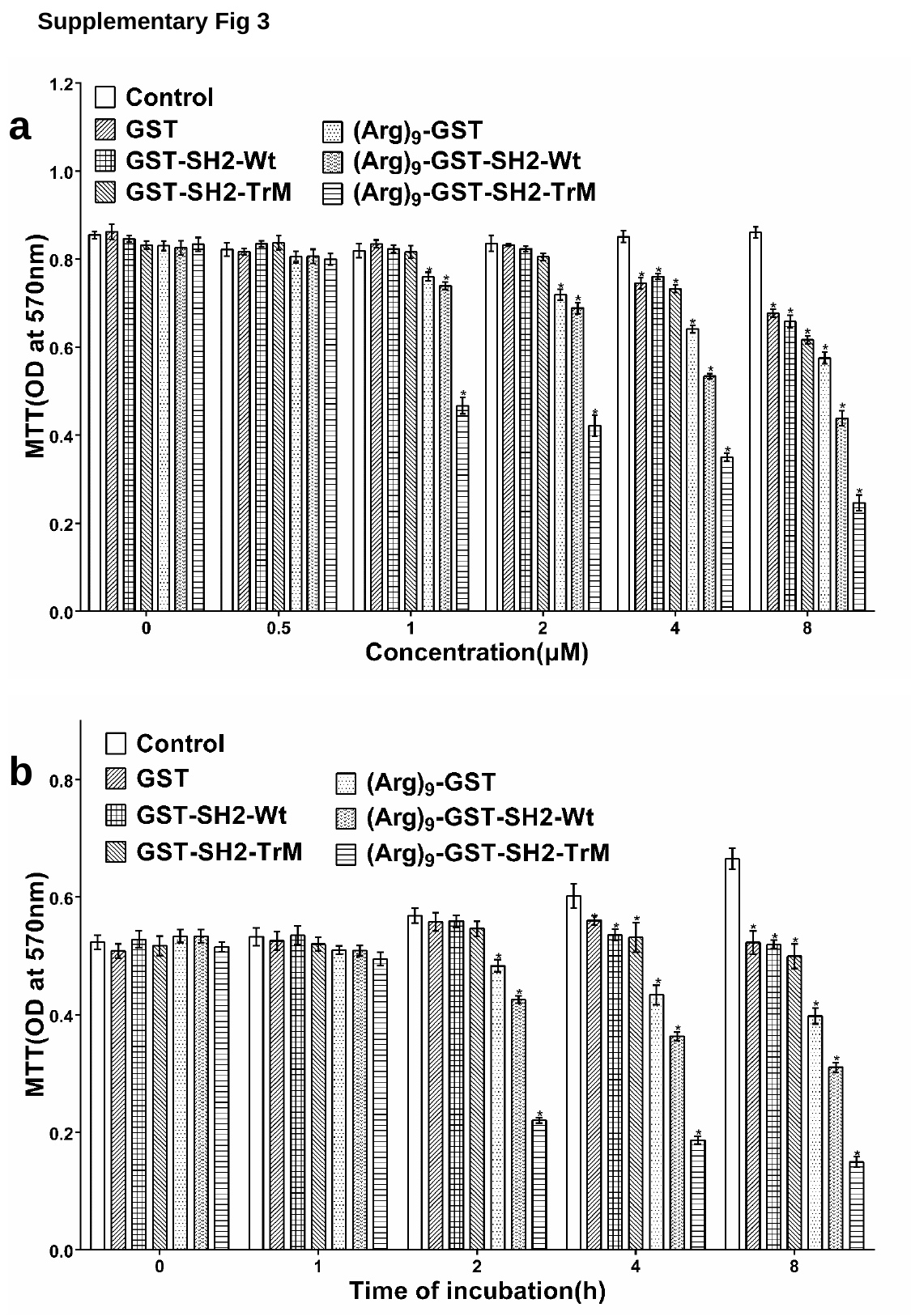

Supplementary Fig 3
a
b

Supplement: Supplementary file 5 — Figure S3. (Arg)9-GST SH2 TrM inhibited the proliferation of A375 cells. Effects of GST, GST SH2 Wt, GST SH2 TrM, (Arg)9-GST, (Arg)9-GST SH2 Wt and (Arg)9-GST SH2 TrM on the proliferation of A375 cells. Cells were treated with different GST-fused proteins at different concentrations (a) (0.5,1, 2, 4 and 8 μM) for various time (b) (1,2,4 and 8 h) and cell viability was measured by MTT assay (n = 3, *P < 0.05). (PPTX 366 kb) [file 13046_2018_812_MOESM5_ESM.pptx]

## Slide 1
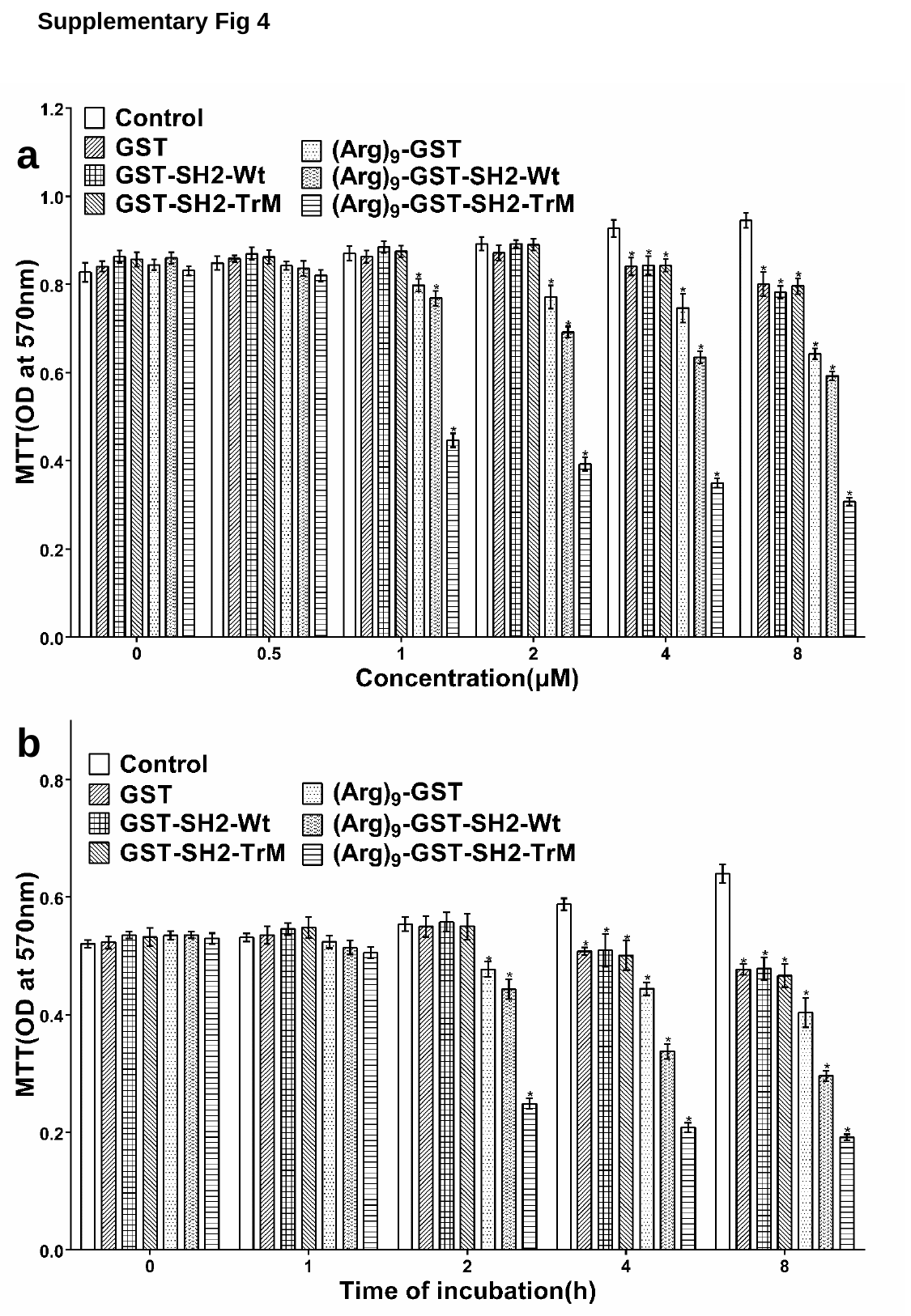

Supplementary Fig 4
a
b

Supplement: Supplementary file 6 — Figure S4. (Arg)9-GST SH2 TrM inhibited the proliferation of A375/DDP cells. Effects of GST, GST SH2 Wt, GST SH2 TrM, (Arg)9-GST, (Arg)9-GST SH2 Wt and (Arg)9-GST SH2 TrM on the proliferation of A375/DDP cells. Cells were treated with different GST-fused proteins at different concentrations (a) (0.5, 1, 2, 4 and 8 μM) for various time (b) (1,2,4 and 8 h) and cell viability was measured by MTT assay (n = 3, *P < 0.05). (PPTX 373 kb) [file 13046_2018_812_MOESM6_ESM.pptx]

## Slide 1
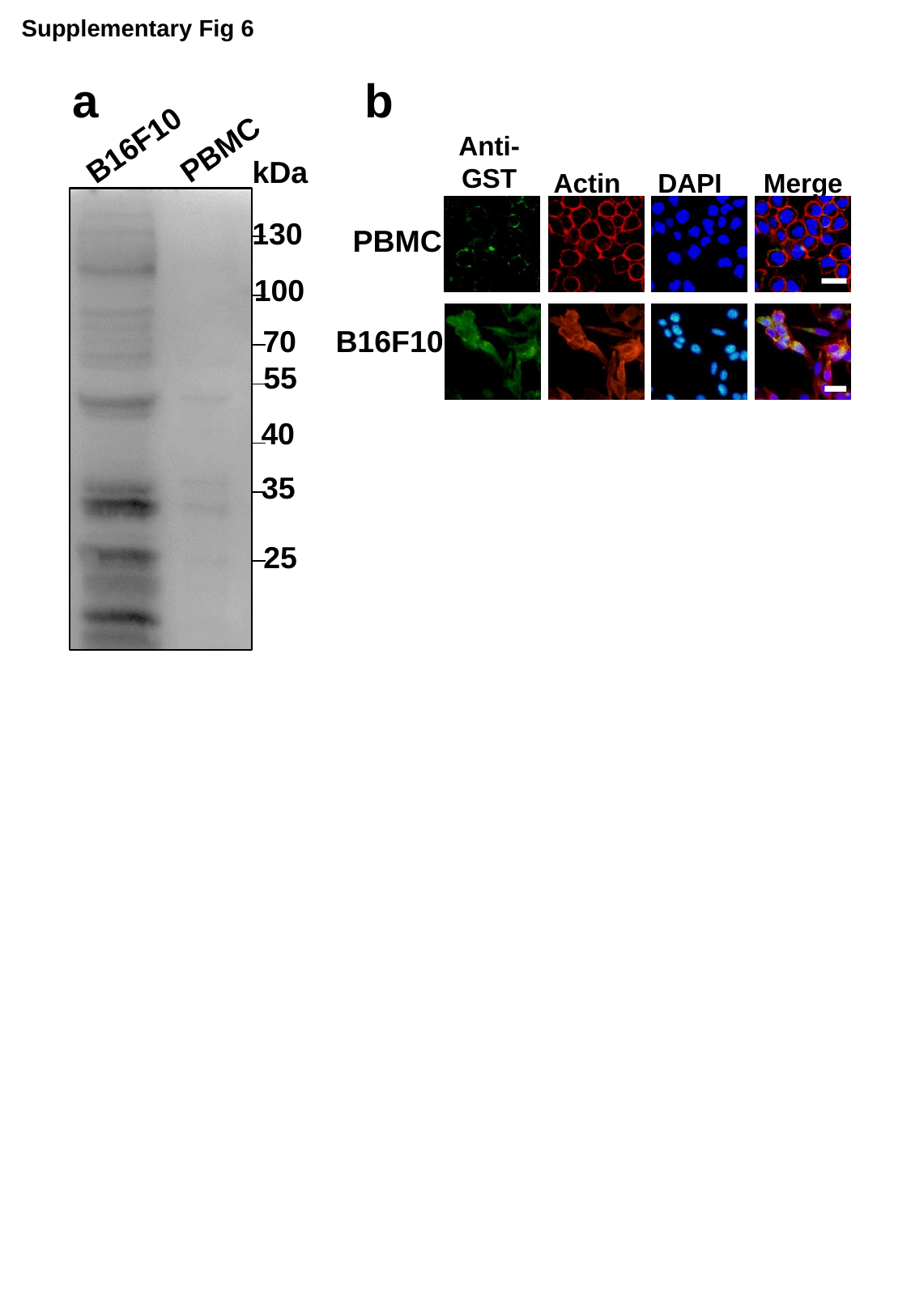

Supplementary Fig 6
a
b
B16F10
PBMC
kDa
130
100
70
55
40
35
25
Anti-
GST
Actin
DAPI
Merge
PBMC
B16F10

Supplement: Supplementary file 8 — Figure S6. The penetration and toxicity effects of (Arg)9-GST SH2 TrM on PBMCs. (a) The level of pY proteins was very low in PBMCs compared with B16F10 cells. (b) (Arg)9-GST-SH2 TrM protein could enter into PBMCs, but could not efficiently be enriched by pY proteins in these non-cancerous cells, thus the green signal was observed to be weak. All images shown are representative of at least three independent experiments. (PPTX 329 kb) [file 13046_2018_812_MOESM8_ESM.pptx]

## Slide 1
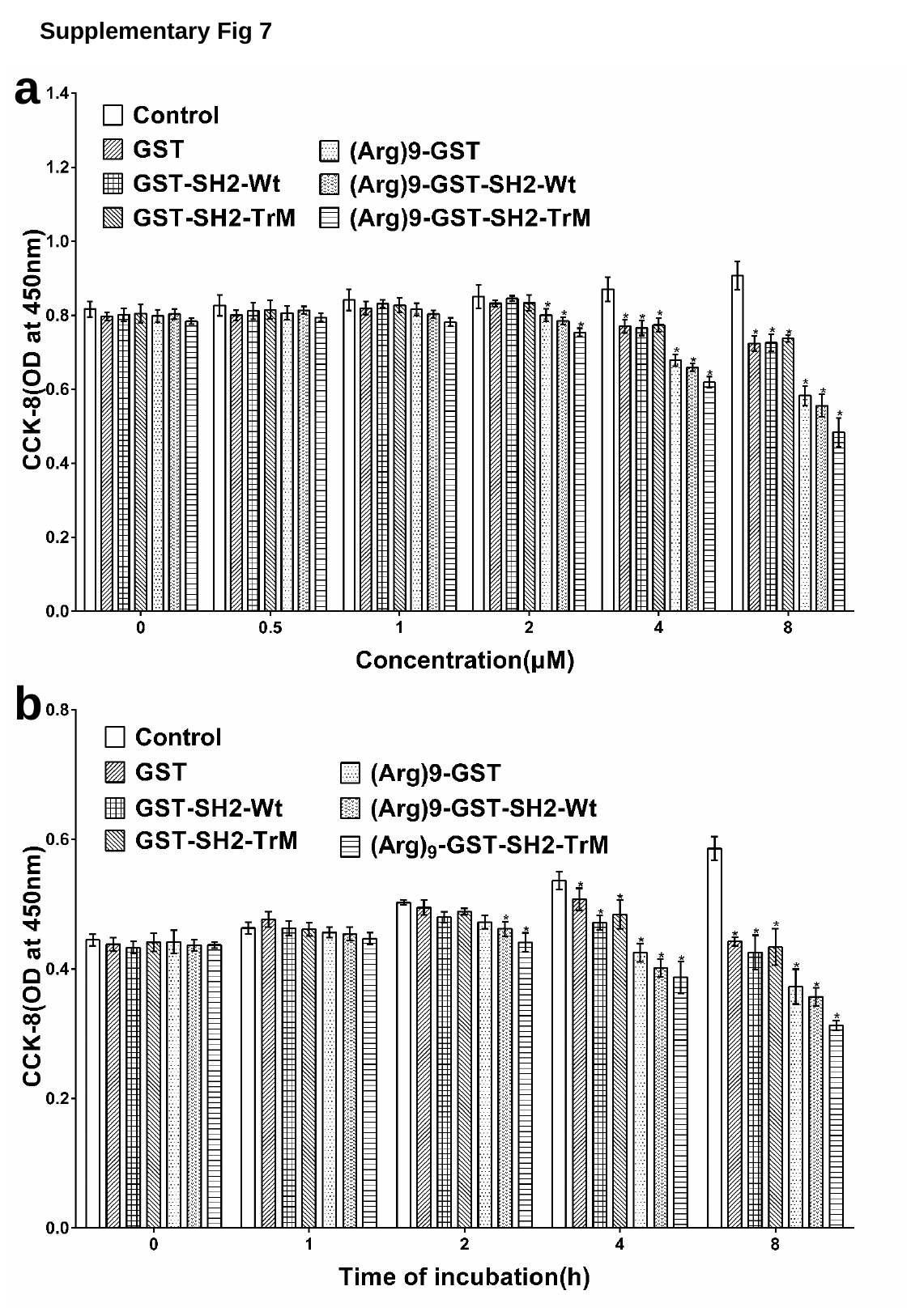

Supplementary Fig 7
a
b

Supplement: Supplementary file 9 — Figure S7. The toxicity effect of (Arg)9-GST SH2 TrM on PBMCs. (Arg)9-GST-SH2 TrM protein resulted in some toxicity effect on PBMCs according to the CCK-8 data, but the effect was moderate as (Arg)9-GST-SH2 TrM protein only specifically recognizing and binding to pY residue, which is low abundance in PBMCs. Cells were treated with different GST-fused proteins at different concentrations (a) (0.5, 1, 2, 4 and 8 μM) for various time (b) (1,2,4 and 8 h) and cell viability was measured by CCK-8 assay (n = 3, *P < 0.05). (PPTX 352 kb) [file 13046_2018_812_MOESM9_ESM.pptx]
