# Supplementary material for: (Arg)9-SH2 superbinder: a novel promising anticancer therapy to melanoma by blocking phosphotyrosine signaling
Source: J Exp Clin Cancer Res. 2018 Jul 5;37:138. doi: 10.1186/s13046-018-0812-5 (PMC6034221; doi:10.1186/s13046-018-0812-5)
Supplement: Supplementary file 7 — Figure S5. Effects of (Arg)9-GST SH2 TrM on JAK/STAT, MAPK/ERK and PI3K/AKT pathways of A375 and A375/DDP cells. Variant levels of phosphorylated and total ERK, AKT (a) and STAT3 (b) from A375 and A375/DDP cells were detected by Western Blot. Cells were treated with or without EGF and (Arg)9-GST SH2 TrM before harvesting. Data shown are representative of three independent experiments. (PPTX 223 kb) [file 13046_2018_812_MOESM7_ESM.pptx]

## Slide 1
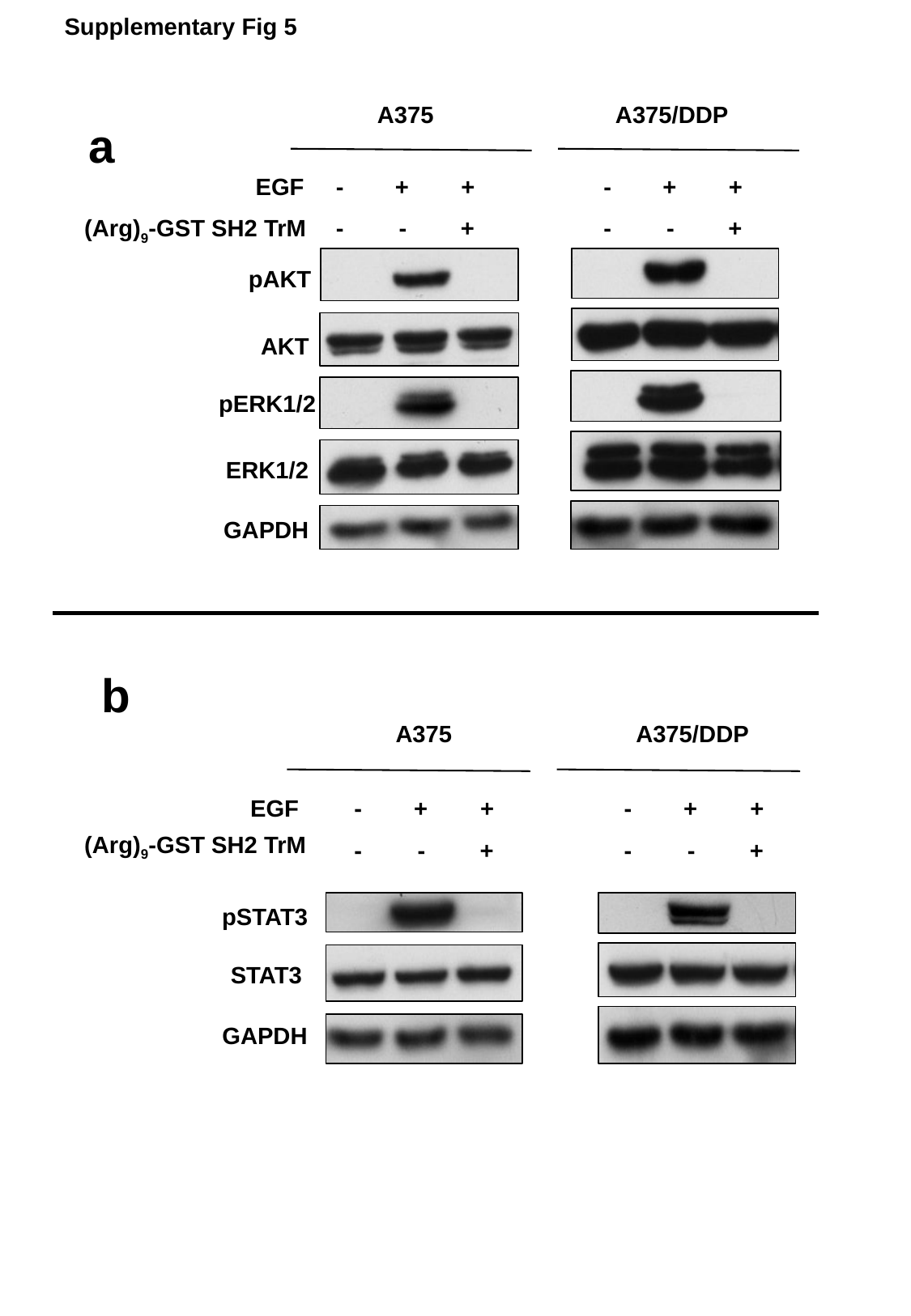

Supplementary Fig 5
A375
A375/DDP
a
EGF
-
+
+
-
-
+
-
+
+
-
-
+
(Arg)9-GST SH2 TrM
pAKT
AKT
pERK1/2
ERK1/2
GAPDH
b
A375
A375/DDP
EGF
-
+
+
-
-
+
-
+
+
-
-
+
(Arg)9-GST SH2 TrM
pSTAT3
STAT3
GAPDH
